# Estimating the average daily rainfall in Thailand using confidence intervals for the common mean of several delta-lognormal distributions

**DOI:** 10.7717/peerj.10758

**Published:** 2021-01-22

**Authors:** Patcharee Maneerat, Sa-Aat Niwitpong

**Affiliations:** 1 Department of Mathematics, Faculty of Science and Technology, Uttaradit Rajabhat University, Uttaradit, Thailand; 2 Department of Applied Statistics, Faculty of Applied Science, King Mongkut’s University of Technology North Bangkok, Bangkok, Thailand

**Keywords:** Agriculture, Bayesian approach, MOVER, Natural rainfall, Vague prior, Variance

## Abstract

The daily average natural rainfall amounts in the five regions of Thailand can be estimated using the confidence intervals for the common mean of several delta-lognormal distributions based on the fiducial generalized confidence interval (FGCI), large sample (LS), method of variance estimates recovery (MOVER), parametric bootstrap (PB), and highest posterior density intervals based on Jeffreys’ rule (HPD-JR) and normal-gamma-beta (HPD-NGB) priors. Monte Carlo simulation was conducted to assess the performance in terms of the coverage probability and average length of the proposed methods. The numerical results indicate that MOVER and PB provided better performances than the other methods in a variety of situations, even when the sample case was large. The efficacies of the proposed methods were illustrated by applying them to real rainfall datasets from the five regions of Thailand.

## Introduction

Approximately 82.2% of Thailand’s cultivated land area depends on natural rainfall (Supasod, 2006), thereby indicating its importance for Thai agriculture. However, it is a natural phenomenon with a significant level of uncertainty that can cause natural disasters such as droughts, floods, and landslides. In many countries around the world, extreme rainfall events have been increasing in frequency and duration. On December 5, 2017, Storm Desmond led to heavy rainfall causing flooding in northern England, Southern Scotland, and Ireland (Otto & Oldenborgh, 2017). On July 6–7, 2018, extreme rainfall events such as floods and landslides affected over 5,000 houses, and approximately 1.9 million people in Japan were evacuated from the at-risk area (Oldenborgh, 2018). In mid-September 2019, the amount of rainfall was extreme during Tropical Storm Imelda in Southeast Texas, USA, where over 1,000 people were affected by large-scale flooding and there were five deaths (Oldenborgh et al., 2019). Thus, it is necessary to assess how rainfall varies in each region of a country on a daily basis. Due to the climate pattern and meteorological conditions, Thailand is commonly separated into five regions: northern, northeastern, central, eastern, and southern. The rainfall in each region varies widely due to both location and seasonality. Importantly, Thailand’s rainfall data include many zeros with probability *δ* > 0 and positive right-skewed data following a lognormal distribution for the remainder of the probability. Thus, applying a delta-lognormal distribution (Aitchison, 1955) is appropriate.

The mean is a measure of the center of a set of observations (Casella & Berger, 2002) that can be used in statistical inference, while functions of the mean such as the ratio or difference between two means can also be used. These parameters have been applied in many research areas, such as medicine, fish stocks, pharmaceutics, and climatology. For example, they have been used for hypothesis testing of the effect of race on the average medical costs between African American and Caucasian patients with type I diabetes (Zhou, Gao & Hui, 1997), to estimate the mean charges for diagnostic tests on patients with unstable chronic medical conditions (Zhou & Tu, 2000; Tian, 2005; Tian & Wu, 2007; Li, Zhou & Tian, 2013), to estimate the maximum alcohol concentration in men in an alcohol interaction study (Tian & Wu, 2007; Krishnamoorthy & Oral, 2015), to estimate the mean red cod density around New Zealand as an indication of fish abundance (Fletcher, 2008; Wu & Hsieh, 2014), and to estimate the mean of the monthly rainfall totals to compare rainfall in Bloemfontein and Kimberley in South African (Harvey & van der Merwe, 2012).

In practice, the mean has been widely used in many fields, as mentioned before. When independent samples are recorded from several situations, then the common mean is of interest when studying more than one population. Many researchers have investigated methods for constructing confidence interval (CIs) for the common mean of several distributions. For example, Fairweather (1972) proposed a linear combination of Student’s t to construct CIs for the common mean of several normal distributions. Jordan & Krishnamoorthy (1996) solved the problem of CIs for the common mean under unknown and unequal variances based on Student’s t and independent F variables from several normal populations. Krishnamoorthy & Mathew (2003) presented the generalized CI (GCI) and compared it with the CIs constructed by Fairweather (1972), and Jordan & Krishnamoorthy (1996). Later, Lin & Lee (2005) developed a GCI for the common mean of several normal populations. Tian & Wu (2007) provided CIs for the common mean of several lognormal populations using the generalized variable approach, which was shown to be consistently better than the large sample (LS) approach. Lin & Wang (2013) studied the modification of the quadratic method to make inference via hypothesis testing and interval estimation for several lognormal means. Krishnamoorthy & Oral (2015) proposed the method of variance estimates recovery (MOVER) approach for the common mean of lognormal distributions.

As mentioned earlier, many researchers have developed CIs for the common mean of several normal and lognormal distributions. However, there has not yet been an investigation of statistical inference using the common mean of several delta-lognormal distributions. Since the common mean is used to study more than one population, the average precipitation in the five regions in Thailand can be estimated using it as there is an important need to estimate the daily rainfall trends in these regions. Furthermore, the daily rainfall records from the five regions in Thailand satisfy the assumptions for a delta-lognormal distribution. Herein, CIs for the common mean of several delta-lognormal models based on the fiducial GCI (FGCI), LS, MOVER, parametric bootstrap (PB), and highest posterior density (HPD) intervals based on Jeffreys’ rule (HPD-JR) and normal-gamma-beta (HPD-NGB) priors are proposed. The outline of this article is as follows. The ideas behind the proposed methods are detailed in the Methods section. Numerical computations are reported in ‘Simulation Studies and Results’. In ‘An Empirical Application’, the daily natural rainfall records of the five regions in Thailand are used to illustrate the efficacy of the methods. Finally, the paper is ended with a discussion and conclusions.

## Methods

Let *W*_*ij*_ = (*W*_*i*1_, *W*_*i*2_, …, *W*_*in*_*i*__) be random samples drawn from a delta-lognormal distribution, for *i* = 1, 2, …, *k* and *j* = 1, 2, .., *n*_*i*_. There are three parameters in this distribution: the mean *μ*_*i*_, variance ${\sigma }_{i}^{2}$ and the probability of obtaining a zero observation *δ*_*i*_. The distribution of *W*_*ij*_ is given by

(1) \begin{eqnarray*}H({w}_{ij};{\mu }_{i},{\sigma }_{i}^{2},{\delta }_{i})= \left\{ \begin{array}{@{}ll@{}} \displaystyle {\delta }_{i} &\displaystyle ;{w}_{ij}=0\\ \displaystyle {\delta }_{i}+(1-{\delta }_{i})G({w}_{ij};{\mu }_{i},{\sigma }_{i}^{2}) &\displaystyle ;{w}_{ij}\gt 0 \end{array} \right. \end{eqnarray*}

where $G({w}_{ij};{\mu }_{i},{\sigma }_{i}^{2})$ is a lognormal distribution function, denoted as $LN({\mu }_{i},{\sigma }_{i}^{2})$ such that $\ln {W}_{ij}\sim N({\mu }_{i},{\sigma }_{i}^{2})$. The number of zeros has a binomial distribution ${n}_{i(0)}=# \left\{ j:{w}_{ij}=0 \right\} \sim B({n}_{i},{\delta }_{i})$. The population mean of *W*_*ij*_ is given by

(2) \begin{eqnarray*}{\vartheta }_{i}=(1-{\delta }_{i})\exp \nolimits \left( {\mu }_{i}+ \frac{{\sigma }_{i}^{2}}{2} \right) \end{eqnarray*}

The unbiased estimates of ${\mu }_{i},{\sigma }_{i}^{2}$, and *δ*_*i*_ are ${\hat {\mu }}_{i}={n}_{i(1)}^{-1}{\sum }_{j:{w}_{ij}\gt 0}\ln {W}_{ij}$, ${\hat {\sigma }}_{i}^{2}=({n}_{i(1)}-1)^{-1}{\sum }_{j:{w}_{ij}\gt 0}{ \left[ \ln {W}_{ij}-{\hat {\mu }}_{i} \right] }^{2}$, and ${\hat {\delta }}_{i}={n}_{i(0)}/{n}_{i}$, respectively, where *n*_*i*_ = *n*_*i*(0)_ + *n*_*i*(1)_; ${n}_{i(1)}=# \left\{ j:{w}_{ij}\gt 0 \right\} $. Suppose that the delta-lognormal mean in Eq. (2) for all *k* populations are the same, then according to Tian & Wu (2007) and Krishnamoorthy & Oral (2015), the common delta-lognormal mean is defined as

(3) \begin{eqnarray*}\vartheta =(1-{\delta }_{i})\exp \nolimits \left( {\mu }_{i}+ \frac{{\sigma }_{i}^{2}}{2} \right) .\end{eqnarray*}

For the *i*th sample, the estimates of *ϑ*_*i*_ are ${\hat {\vartheta }}_{i}^{\ast }=(1-{\hat {\delta }}_{i})\exp ({\hat {\mu }}_{i}+ \frac{{\hat {\sigma }}_{i}^{2}}{2} )$ which contains the unbiased estimates ${\hat {\mu }}_{i}$, ${\hat {\sigma }}_{i}^{2}$ and ${\hat {\delta }}_{i}$. According to Longford (2009), the expected value of ${\hat {\vartheta }}_{i}^{\ast }$ is derived as

(4) \begin{eqnarray*}E[{\hat {\vartheta }}_{i}^{\ast }]& = \left[ 1-E({\hat {\delta }}_{i}) \right] E \left[ \exp \nolimits \left\{ {\hat {\mu }}_{i}+ \frac{{\hat {\sigma }}_{i}^{2}}{2} \right\} \right] \end{eqnarray*}

(5) \begin{eqnarray*}=(1-{\delta }_{i})\exp \nolimits \left( {\mu }_{i}+ \frac{{\sigma }_{i}^{2}}{{n}_{i(1)}} \right) { \left( \frac{{l}_{i}}{{l}_{i}-{\sigma }_{i}^{2}} \right) }^{{l}_{i}/2}\end{eqnarray*}

where ${\hat {\delta }}_{i}\sim N({\delta }_{i}, \frac{{\delta }_{i}(1-{\delta }_{i})}{{n}_{i}} )$ as *n*_*i*_ → ∞, $E[\exp ({\hat {\mu }}_{i})]=\exp \left( {\mu }_{i}+ \frac{{\sigma }_{i}^{2}}{2{n}_{i(1)}} \right) $ and *E*[exp(*c*_*i*_*Y*_*i*_)] = (1 − 2*c*_*i*_)^−*l*∕2^; ${Y}_{i}={l}_{i} \frac{{\hat {\sigma }}_{i}^{2}}{{\sigma }_{i}^{2}} \sim {\chi }_{{l}_{i}}^{2}$ and ${c}_{i}= \frac{{\sigma }_{i}^{2}}{2{l}_{i}} $, ${\hat {\sigma }}_{i}^{2}=({n}_{i(1)}-1)^{-1}{\mathop{\sum }\nolimits }_{j=1}^{{n}_{i(1)}}{ \left[ \ln ({W}_{ij})-{\hat {\mu }}_{i} \right] }^{2}$. If $ \frac{{l}_{i}-{\sigma }_{i}^{2}}{{l}_{i}} =\exp \left[ \frac{-2{\sigma }_{i}^{2}}{{l}_{i}} ( \frac{1}{2} - \frac{1}{2{n}_{i(1)}} ) \right] $, then we can obtain that

(6) \begin{eqnarray*}E[{\hat {\vartheta }}_{i}^{\ast }]& =(1-{\delta }_{i})\exp \nolimits \left( {\mu }_{i}+ \frac{{\sigma }_{i}^{2}}{2{n}_{i(1)}} \right) { \left\{ \exp \nolimits \left[ \frac{-2{\sigma }_{i}^{2}}{{l}_{i}} ( \frac{1}{2} - \frac{1}{2{n}_{i(1)}} ) \right] \right\} }^{-{l}_{i}/2}\nonumber\\\displaystyle & =(1-{\delta }_{i})\exp \nolimits \left( {\mu }_{i}+ \frac{{\sigma }_{i}^{2}}{2} \right) .\end{eqnarray*}

According to Aitchison & Brown (1963), the Aitchison estimate of *ϑ*_*i*_ is expressed as

(7) \begin{eqnarray*}{\hat {\vartheta }}_{i}^{(Ait)}= \left\{ \begin{array}{@{}ll@{}} \displaystyle 0 &\displaystyle ;{n}_{i(1)}=0\\ \displaystyle {w}_{i1}/{n}_{i} &\displaystyle ;{n}_{i(1)}=1\\ \displaystyle (1-\hat {{\delta }_{i}})\exp \nolimits (\hat {{\mu }_{i}}){\psi }_{{n}_{i(1)}}( \frac{{\hat {\sigma }}_{i}^{2}}{2} ) &\displaystyle ;{n}_{i(1)}\gt 1 \end{array} \right. \end{eqnarray*}

where *ψ*_*a*_(*b*) is a Bessel function defined as

(8) \begin{eqnarray*}{\psi }_{a}(b)=1+ \frac{(a-1)b}{a} + \frac{(a-1)^{3}}{{a}^{2}2{!}} \frac{{b}^{2}}{a+1} + \frac{(a-1)^{5}}{{a}^{3}3{!}} \frac{{b}^{3}}{(a+1)(a+3)} +...\end{eqnarray*}

To investigate the unbiased estimate ${\hat {\vartheta }}_{i}^{(Ait)}$, the expected value is

\begin{eqnarray*}E \left[ {\hat {\vartheta }}_{i}^{(Ait)} \right] & =\sum _{j=1}^{{n}_{i}}P({n}_{i(1)}=j)E \left[ \hat {{\vartheta }_{i}}{|}{n}_{i(1)}=j \right] \nonumber\\\displaystyle & =0+P({n}_{i(1)}=1)E \left[ {w}_{i1}/{n}_{i} \right] +\sum _{j=2}^{{n}_{i}}P({n}_{i(1)}=j)E \left[ \hat {{\vartheta }_{i}}{|}{n}_{i(1)}=j \right] \nonumber\\\displaystyle & =P({n}_{i(1)}=1) \frac{\exp \nolimits ({\mu }_{i}+ \frac{{\sigma }_{i}^{2}}{2} )}{{n}_{i}} +\sum _{j=2}^{{n}_{i}}P({n}_{i(1)}=j)E \left[ \frac{{n}_{i(1)}}{{n}_{i}} \exp \nolimits ({\mu }_{i}+ \frac{{\sigma }_{i}^{2}}{2} ){|}{n}_{i(1)}=j \right] \nonumber\\\displaystyle & =\sum _{j=0}^{{n}_{i}}P({n}_{i(1)}=j)E \left[ \frac{{n}_{i(1)}}{{n}_{i}} \exp \nolimits ({\mu }_{i}+ \frac{{\sigma }_{i}^{2}}{2} ){|}{n}_{i(1)}=j \right] \nonumber\\\displaystyle & =E \left[ \frac{{n}_{i(1)}}{{n}_{i}} \exp \nolimits ({\mu }_{i}+ \frac{{\sigma }_{i}^{2}}{2} ) \right] \nonumber\\\displaystyle & =(1-{\delta }_{i})\exp \nolimits ({\mu }_{i}+ \frac{{\sigma }_{i}^{2}}{2} ). \end{eqnarray*}

According to Shimizu & Iwase (1981), the uniformly minimum variance unbiased (UMVU) estimate of *ϑ*_*i*_ is

(9) \begin{eqnarray*}{\hat {\vartheta }}_{i}^{(Shi)}= \left\{ \begin{array}{@{}cc@{}} \displaystyle 0&\displaystyle ;{n}_{i(1)}\lt 1\\ \displaystyle \frac{{n}_{i(1)}}{{n}_{i}} \exp \nolimits ({\hat {\mu }}_{i})_{0}{F}_{1} \left( \frac{{n}_{i(1)}-1}{2} ; \frac{{n}_{i(1)}-1}{4{n}_{i(1)}} {S}_{i}^{2} \right) &\displaystyle ;{n}_{i(1)}\geq 1 \end{array} \right. \end{eqnarray*}

where ${S}_{i}^{2}={\mathop{\sum }\nolimits }_{j=1}^{{n}_{i(1)}}{ \left[ \ln ({W}_{ij})-{\hat {\mu }}_{i} \right] }^{2}$ and ${}_{0}{F}_{1}(a;z)={\mathop{\sum }\nolimits }_{m=0}^{\infty } \frac{{z}^{m}}{(a)_{m}m{!}} $;

(10) \begin{eqnarray*}(a)_{m}= \left\{ \begin{array}{@{}cc@{}} \displaystyle 1&\displaystyle ;m=0\\ \displaystyle a(a+1)...(a+m-1)&\displaystyle ;m\geq 1 \end{array} \right. \end{eqnarray*}

From Kunio (1983), $E \left[ {\text{}}_{0}{F}_{1} \left( \frac{{n}_{i(1)}-1}{2} ; \frac{a}{2} {S}_{i}^{2} \right) \right] =\exp (a{\sigma }^{2})$ is obtained, then

(11) \begin{eqnarray*}E \left[ {\hat {\vartheta }}_{i}^{(Shi)} \right] & =E \left[ \frac{{n}_{i(1)}}{n} \exp \nolimits ({\hat {\mu }}_{i})_{0}{F}_{1} \left( \frac{{n}_{i(1)}-1}{2} , \frac{{n}_{i(1)}-1}{4{n}_{i(1)}} {S}_{i}^{2} \right) \right] \nonumber\\\displaystyle & = \frac{{n}_{i}(1-{\delta }_{i})}{{n}_{i}} \exp \nolimits \left[ {\mu }_{i}+ \frac{{\sigma }_{i}^{2}}{2{n}_{i(1)}} \right] \exp \nolimits \left[ \frac{{n}_{i(1)}-1}{2{n}_{i(1)}} {\sigma }_{i}^{2} \right] \nonumber\\\displaystyle & =(1-{\delta }_{i})\exp \nolimits \left( {\mu }_{i}+ \frac{{\sigma }_{i}^{2}}{2} \right) \end{eqnarray*}

where *E*(*n*_*i*(1)_) = *n*_*i*_(1 − *δ*_*i*_). The asymptotic variance of ${\hat {\vartheta }}_{i}^{(Shi)}$ is given by

\begin{eqnarray*}& & Var \left[ {\hat {\vartheta }}_{i}^{(Shi)} \right] =\exp \nolimits (2{\mu }_{i}+{\sigma }_{i}^{2}) \left[ \frac{1}{{n}_{i}^{2}} \sum _{j=1}^{{n}_{i}} \left( {{n}_{i}\atop j} \right) (1-{\delta }_{i})^{j}{\delta }^{{n}_{i}-j}{j}^{2}\exp \nolimits \left( \frac{{\sigma }_{i}^{2}}{j} \right) \right. \end{eqnarray*}

\begin{eqnarray*}& & \left. {\text{}}_{0}{F}_{1} \left( \frac{j-1}{2} ; \frac{(j-1)^{2}}{4{j}^{2}} {\sigma }_{i}^{4} \right) -(1-{\delta }_{i})^{2} \right] \end{eqnarray*}

(12) \begin{eqnarray*}& & = \frac{\exp \nolimits (2{\mu }_{i}+{\sigma }_{i}^{2})}{{n}_{i}} \left[ {\delta }_{i}(1-{\delta }_{i})+ \frac{1}{2} (1-{\delta }_{i})(2{\sigma }_{i}^{2}+{\sigma }_{i}^{4}) \right] +O({n}^{-2}).\end{eqnarray*}

Actually, ${\psi }_{{n}_{i(1)}}( \frac{{\hat {\sigma }}_{i}^{2}}{2} ){=}_{0}{F}_{1} \left( \frac{{n}_{i(1)}-1}{2} ; \frac{{n}_{i(1)}-1}{4{n}_{i(1)}} {S}_{i}^{2} \right) $ such that ${\hat {\vartheta }}_{i}^{(Shi)}$ and ${\hat {\vartheta }}_{i}^{(Ait)}$ are the unbiased estimates of *ϑ*_*i*_ under different ideas, although their variances are the same i.e., $Var \left[ {\hat {\vartheta }}_{i}^{(Shi)} \right] =Var \left[ {\hat {\vartheta }}_{i}^{(Ait)} \right] $. Using $\hat {{\mu }_{i}},{\hat {\sigma }}_{i}^{2}$, and $\hat {{\delta }_{i}}$ from the samples, the estimated delta-lognormal mean ${\hat {\vartheta }}_{i}^{(Ait)}$ and variance of ${\hat {\vartheta }}_{i}^{(Ait)}$ are obtained. The following methods are the detailed construction of the CIs for the common delta-lognormal mean.

### Fiducial generalized confidence interval

Fiducial inference was introduced by Fisher (1930). Fisher’s fiducial argument was used to develop a generalized fiducial recipe that could be extended to the application of fiducial ideas (Hannig, 2009). The concept of the fiducial interval has been advanced by the idea of the generalized pivotal quantity (GPQ) such that it is directly used to apply for generalized inference. Later, Hannig, Iyer & Patterson (2006) argued that a subclass of GPQs, the fiducial GPQ (FGPQ), provides a framework that shows the connection between a distribution and a parameter. Recall that ${\hat {\mu }}_{i}\sim N({\mu }_{i},{\sigma }_{i}^{2}/{n}_{i(1)})$ and $({n}_{i(1)}-1){\hat {\sigma }}_{i}^{2}/{\sigma }_{i}^{2}\sim {\chi }_{{n}_{i(1)}-1}^{2}$ are the independent random variables. The structure functions of ${\hat {\mu }}_{i}$ and ${\hat {\sigma }}_{i}^{2}$ are

(13) \begin{eqnarray*}{\hat {\mu }}_{i}={\mu }_{i}+{V}_{i}\sqrt{ \frac{{\sigma }_{i}^{2}}{{n}_{i(1)}} } \text{and} {\hat {\sigma }}_{i}^{2}= \frac{{\sigma }_{i}^{2}{U}_{i}}{{n}_{i(1)}-1} \end{eqnarray*}

which are the function of *V*_*i*_ and *U*_*i*_, respectively, where *V*_*i*_ ∼ *N*(0, 1) and ${U}_{i}\sim {\chi }_{{n}_{i(1)}-1}^{2}$. Given the observed values, the estimates ${\hat {\mu }}_{i}$ and ${\hat {\sigma }}_{i}^{2}$ can be obtained, and the unique solution of $ \left( {\hat {\mu }}_{i},{\hat {\sigma }}_{i}^{2} \right) = \left( {\mu }_{i}+{V}_{i}\sqrt{ \frac{{\sigma }_{i}^{2}}{{n}_{i(1)}} }, \frac{{\sigma }_{i}^{2}{U}_{i}}{{n}_{i(1)}-1} \right) $ becomes

(14) \begin{eqnarray*}{\mu }_{i}={\hat {\mu }}_{i}-{V}_{i} \frac{{\hat {\sigma }}_{i}}{\sqrt{{n}_{i(1)}}} \sqrt{ \frac{{n}_{i(1)}-1}{{U}_{i}} }, {\sigma }_{i}^{2}= \frac{({n}_{i(1)}-1){\hat {\sigma }}_{i}^{2}}{{U}_{i}} .\end{eqnarray*}

The respective FGPQs of *μ*_*i*_ and ${\sigma }_{i}^{2}$ are

(15) \begin{eqnarray*}{G}_{{\mu }_{i}}& ={\hat {\mu }}_{i}-{V}_{i}^{\ast } \frac{{\hat {\sigma }}_{i}}{\sqrt{{n}_{i(1)}}} \sqrt{ \frac{{n}_{i(1)}-1}{{U}_{i}^{\ast }} }\end{eqnarray*}

(16) \begin{eqnarray*}{G}_{{\sigma }_{i}^{2}}& = \frac{({n}_{i(1)}-1){\hat {\sigma }}_{i}^{2}}{{U}_{i}^{\ast }} \end{eqnarray*}

where ${V}_{i}^{\ast }$ and ${U}_{i}^{\ast }$ are independent copies of *V*_*i*_ and *U*_*i*_, respectively. Hasan & Krishnamoorthy (2018) developed the FGPQ of *δ*_*i*_ using a beta distribution as ${G}_{{\delta }_{i}^{{^{\prime}}}}\sim Beta({\alpha }_{i},{\beta }_{i})$; *α*_*i*_ = *n*_*i*(1)_ + 0.5 and *β*_*i*_ = *n*_*i*(0)_ + 0.5. The FGPQ of *ϑ* based on *k* individual samples is

(17) \begin{eqnarray*}{G}_{\vartheta }= \frac{\sum _{i=1}^{k}{G}_{{w}_{i}}{G}_{{\vartheta }_{i}}}{\sum _{i=1}^{k}{G}_{{w}_{i}}} \end{eqnarray*}

where ${G}_{{\vartheta }_{i}}={G}_{{\delta }_{i}^{{^{\prime}}}}\exp ({G}_{{\mu }_{i}}+{G}_{{\sigma }_{i}^{2}}/2)$, ${G}_{{w}_{i}}=1/{G}_{Var \left[ {\hat {\vartheta }}_{i}^{(Ait)} \right] }$, and ${G}_{Var \left[ {\hat {\vartheta }}_{i}^{(Ait)} \right] }=\exp (2{G}_{{\mu }_{i}}+{G}_{{\sigma }_{i}^{2}}) \left[ {G}_{{\delta }_{i}^{{^{\prime}}}}(1-{G}_{{\delta }_{i}^{{^{\prime}}}})+ \frac{1}{2} {G}_{{\delta }_{i}^{{^{\prime}}}}(2{G}_{{\sigma }_{i}^{2}}+{G}_{{\sigma }_{i}^{4}}) \right] /{n}_{i}$. Thus, the 100(1 − *ζ*)% FGCI for *ϑ* is

(18) \begin{eqnarray*}C{I}_{\vartheta }^{(fgci)}=[{L}_{\vartheta }^{(fgci)},{U}_{\vartheta }^{(fgci)}]=[{G}_{\vartheta }(\zeta /2),{G}_{\vartheta }(1-\zeta /2)]\end{eqnarray*}

where *G*_*ϑ*_(*ζ*) denotes the *ζ*^th^ percentiles of *G*_*ϑ*_. Algorithm 1 shows the computational steps for obtaining the FGCI.

#### Algorithm 1: FGCI

(1) Generate *V*_*i*_ ∼ *N*(0, 1) and ${U}_{i}\sim {\chi }_{{n}_{i(1)}-1}^{2}$ are independent.
(2) Compute the FGPQs *G*_*μ*_*i*__, ${G}_{{\sigma }_{i}^{2}}$ and ${G}_{{\delta }_{i}^{{^{\prime}}}}$.
(3) Compute *G*_*w*_*i*__ and *G*_*ϑ*_*i*__ leading to obtain *G*_*ϑ*_.
(4) Repeat steps 1-3, a number of times, *m* = 2500, compute 95%FGCI for *ϑ*, as given in Eq. (18).

### Large sample interval

Recall that the Aitchitson estimator is ${\hat {\vartheta }}_{i}^{(Ait)}=(1-\hat {{\delta }_{i}})\exp (\hat {{\mu }_{i}}){\psi }_{{n}_{i(1)}}({\hat {\sigma }}_{i}^{2}/2)$ and the variance of ${\hat {\vartheta }}_{i}^{(Ait)}$ is $Var \left[ {\hat {\vartheta }}_{i}^{(Ait)} \right] =\exp (2{\mu }_{i}+{\sigma }_{i}^{2}) \left[ {\delta }_{i}(1-{\delta }_{i})+ \frac{1}{2} (1-{\delta }_{i})(2{\sigma }_{i}^{2}+{\sigma }_{i}^{4}) \right] /{n}_{i}$. The approximated variance is obtained by replacing ${\hat {\mu }}_{i}$, ${\hat {\sigma }}_{i}^{2}$ and ${\hat {\delta }}_{i}$. The pooled estimate of *ϑ*_*i*_ is given by

(19) \begin{eqnarray*}\hat {\vartheta }= \frac{\sum _{i=1}^{k}{w}_{i}{\hat {\vartheta }}_{i}^{(Ait)}}{\sum _{i=1}^{k}{w}_{i}} \end{eqnarray*}

where ${w}_{i}=1/\widehat{Var} \left[ {\hat {\vartheta }}_{i}^{(Ait)} \right] $. Hence, the 100(1 − *ζ*)% LS interval for *ϑ* is obtained as

(20) \begin{eqnarray*}C{I}_{\vartheta }^{(ls)}=[{L}_{\vartheta }^{(ls)},{U}_{\vartheta }^{(ls)}]= \left[ \hat {\vartheta }-{z}_{1- \frac{\zeta }{2} }\sqrt{1/\sum _{i=1}^{k}{w}_{i}},\hat {\vartheta }+{z}_{1- \frac{\zeta }{2} }\sqrt{1/\sum _{i=1}^{k}{w}_{i}} \right] \end{eqnarray*}

where *z*_*ζ*_ denotes the *ζ*^th^ percentiles of standard normal *N*(0, 1). The LS interval can be estimated easily via ‘Algorithm 2’.

#### Algorithm 2: LS

(1) Compute ${\hat {\vartheta }}_{i}^{(Ait)}$ and $\widehat{Var} \left[ {\hat {\vartheta }}_{i}^{(Ait)} \right] $.
(2) Compute $\hat {\vartheta }$.
(3) Compute 95%LS interval for *ϑ*, as given in Eq. (20).

### Method of variance estimates recovery

This method produces a closed-form CI that is easy to compute. For this reason, the MOVER CI for the common delta-lognormal mean is considered for *k* individual random samples. The MOVER for a linear combination of *ϑ*_*i*_; i=1 , 2, …, *k* is as follows. Let ${\hat {\vartheta }}_{1},{\hat {\vartheta }}_{2},\ldots ,{\hat {\vartheta }}_{k}$ be independent unbiased estimators of *ϑ*_1_, *ϑ*_2_, …, *ϑ*_*k*_, respectively. In addition, let [*l*_*i*_, *u*_*i*_] stand for the 100(1 − *ζ*)%CI for *ϑ*_*i*_. According to Krishnamoorthy & Oral (2015), the 100(1 − *ζ*)%MOVER for ${\mathop{\sum }\nolimits }_{i=1}^{k}{c}_{i}{\vartheta }_{i}$ is given by

(21) \begin{eqnarray*}C{I}_{\sum _{i=1}^{k}{c}_{i}{\vartheta }_{i}}& =[{L}_{\sum _{i=1}^{k}{c}_{i}{\vartheta }_{i}},{U}_{\sum _{i=1}^{k}{c}_{i}{\vartheta }_{i}}]\nonumber\\\displaystyle & = \left[ \sum _{i=1}^{k}{c}_{i}{\hat {\vartheta }}_{i}-\sqrt{\sum _{i=1}^{k}{c}_{i}^{2}{ \left( {\hat {\vartheta }}_{i}-{l}_{i}^{\ast } \right) }^{2}},\sum _{i=1}^{k}{c}_{i}{\hat {\vartheta }}_{i}+\sqrt{\sum _{i=1}^{k}{c}_{i}^{2}{ \left( {\hat {\vartheta }}_{i}-{u}_{i}^{\ast } \right) }^{2}} \right] \end{eqnarray*}

where ${l}_{i}^{\ast }= \left\{ {\scriptsize \begin{array}{@{}ll@{}} \displaystyle {l}_{i} &\displaystyle ;{c}_{i}\gt 0\\ \displaystyle {u}_{i} &\displaystyle ;{c}_{i}\lt 0 \end{array}} \right. $ and ${u}_{i}^{\ast }= \left\{ {\scriptsize \begin{array}{@{}ll@{}} \displaystyle {u}_{i} &\displaystyle ;{c}_{i}\gt 0\\ \displaystyle {l}_{i} &\displaystyle ;{c}_{i}\lt 0 \end{array}} \right. $. Next, the closed-form CIs for *ϑ*_*i*_ are needed to construct MOVER for *ϑ*. Thus, *ϑ*_*i*_ is log-transformed as

(22) \begin{eqnarray*}\ln \nolimits {\vartheta }_{i}=\ln \nolimits {\delta }_{i}^{\ast }+({\mu }_{i}+{\sigma }_{i}^{2})\end{eqnarray*}

where ${\delta }_{i}^{\ast }=1-{\delta }_{i}$. Let ${\hat {\mu }}_{i}$, and ${\hat {\sigma }}_{i}^{2}$ and ${\hat {\delta }}^{\ast }$ be the unbiased estimates of *μ*_*i*_, ${\sigma }_{i}^{2}$, and *δ*_*i*_, respectively. The MOVER for a single delta-lognormal mean presented by Hasan & Krishnamoorthy (2018), the MOVER for *ϑ*_*i*_ is given by

(23) \begin{eqnarray*}{L}_{{\vartheta }_{i}} & =\exp \nolimits \left\{ \ln \nolimits {\hat {\delta }}_{i}^{\ast }+({\hat {\mu }}_{i}+{\hat {\sigma }}_{i}^{2})-\sqrt{{ \left( \ln \nolimits {\hat {\delta }}_{i}^{\ast }-{l}_{\ln \nolimits {\delta }_{i}^{\ast }} \right) }^{2}+{ \left( {\hat {\mu }}_{i}+{\hat {\sigma }}_{i}^{2}-{l}_{{\mu }_{i}+{\sigma }_{i}^{2}} \right) }^{2}} \right\} {U}_{{\vartheta }_{i}} & =\exp \nolimits \left\{ \ln \nolimits {\hat {\delta }}_{i}^{\ast }+({\hat {\mu }}_{i}+{\hat {\sigma }}_{i}^{2})-\sqrt{{ \left( \ln \nolimits {\hat {\delta }}_{i}^{\ast }-{u}_{\ln \nolimits {\delta }_{i}^{\ast }} \right) }^{2}+{ \left( {\hat {\mu }}_{i}+{\hat {\sigma }}_{i}^{2}-{u}_{{\mu }_{i}+{\sigma }_{i}^{2}} \right) }^{2}} \right\} \end{eqnarray*}

where

\begin{eqnarray*}({l}_{\ln \nolimits {\delta }_{i}^{\ast }},{u}_{\ln \nolimits {\delta }_{i}^{\ast }})& =& \ln \nolimits \left[ \left( {\hat {\delta }}_{i}^{\ast }+ \frac{{T}_{i,\zeta /2}^{2}}{2{n}_{i}} \mp {T}_{i,1-\zeta /2}\sqrt{ \frac{{\hat {\delta }}_{i}^{\ast }(1-{\hat {\delta }}_{i}^{\ast })}{{n}_{i}} + \frac{{T}_{i,\zeta /2}^{2}}{4{n}_{i}^{2}} } \right) /(1+{T}_{i,\zeta /2}^{2}/{n}_{i}) \right] \end{eqnarray*}

(24) \begin{eqnarray*}({l}_{{\mu }_{i}+{\sigma }_{i}^{2}},{u}_{{\mu }_{i}+{\sigma }_{i}^{2}})& =& \left[ ({\hat {\mu }}_{i}+{\hat {\sigma }}_{i}^{2}/2)-{ \left\{ { \left( \frac{{Z}_{i,\zeta /2}{\hat {\sigma }}_{i}^{2}}{{n}_{i(1)}} \right) }^{2}+ \frac{{\hat {\sigma }}_{i}^{4}}{4} { \left( 1- \frac{{n}_{i(1)}-1}{{\chi }_{i,1-\zeta /2,{n}_{i(1)}-1}^{2}} \right) }^{2} \right\} }^{1/2}, \right. \nonumber\\\displaystyle & & \left. ({\hat {\mu }}_{i}+{\hat {\sigma }}_{i}^{2}/2)+{ \left\{ { \left( \frac{{Z}_{i,\zeta /2}{\hat {\sigma }}_{i}^{2}}{{n}_{i(1)}} \right) }^{2}+ \frac{{\hat {\sigma }}_{i}^{4}}{4} { \left( \frac{{n}_{i(1)}-1}{{\chi }_{i,\zeta /2,{n}_{i(1)}-1}^{2}} -1 \right) }^{2} \right\} }^{1/2} \right] .\end{eqnarray*}

Note that both ${T}_{i}=({n}_{i(1)}-{n}_{i}{\delta }^{\ast })/\sqrt{{n}_{i}{\delta }_{i}^{\ast }(1-{\delta }_{i}^{\ast })}\sim ^{d}N(0,1)$, and ${Z}_{i}=({\hat {\mu }}_{i}-{\mu }_{i})/\sqrt{{\hat {\sigma }}_{i}^{2}/{n}_{i(1)}}\sim ^{d}N(0,1)$ are independent random variables. According to Krishnamoorthy & Oral (2015), the 100(1 − *ζ*)% MOVER interval for *ϑ* is

(25) \begin{eqnarray*}C{I}_{\vartheta }^{(mover)} & =[{L}_{\vartheta },{U}_{\vartheta }] & = \left[ \frac{\sum _{i=1}^{k}{w}_{i}{\hat {\vartheta }}_{i}^{(Ait)}}{\sum _{i=1}^{k}{w}_{i}} -\sqrt{ \frac{\sum _{i=1}^{k}{w}_{i}^{2}{ \left( {\hat {\vartheta }}_{i}^{(Ait)}-{L}_{{\vartheta }_{i}} \right) }^{2}}{\sum _{i=1}^{k}{w}_{i}^{2}} }, \right. \nonumber\\\displaystyle & \left. \frac{\sum _{i=1}^{k}{w}_{i}{\hat {\vartheta }}_{i}^{(Ait)}}{\sum _{i=1}^{k}{w}_{i}} -\sqrt{ \frac{\sum _{i=1}^{k}{w}_{i}^{2}{ \left( {\hat {\vartheta }}_{i}^{(Ait)}-{U}_{{\vartheta }_{i}} \right) }^{2}}{\sum _{i=1}^{k}{w}_{i}^{2}} } \right] \end{eqnarray*}

where ${w}_{i}=1/\widehat{Var} \left[ {\hat {\vartheta }}_{i}^{(Ait)} \right] $. ‘Algorithm 3’ describes the steps to construct the MOVER interval.

#### Algorithm 3: MOVER

(1) Compute CIs for $\ln  {\delta }_{i}^{\ast }$ and ${\mu }_{i}+{\sigma }_{i}^{2}$ are $({l}_{\ln {\delta }_{i}^{\ast }},{u}_{\ln {\delta }_{i}^{\ast }})$ and $({l}_{{\mu }_{i}+{\sigma }_{i}^{2}},{u}_{{\mu }_{i}+{\sigma }_{i}^{2}})$, respectively.
(2) Compute MOVER for *ϑ*_*i*_, as given in Eq. (23).
(3) Compute 95%MOVER for *ϑ*, given in Eq. (25).

### Parametric Bootstrap

This is developed from the parametric bootstrap on the common mean of several heterogeneous log-normal distributions, proposed by Malekzadeh & Kharrati-Kopaei (2019). The delta-lognormal mean is transformed by taking the logarithm as

(26) \begin{eqnarray*}{\mu }_{i}=\ln \nolimits \left( \frac{\vartheta }{1-{\delta }_{i}} \right) - \frac{{\sigma }_{i}^{2}}{2} .\end{eqnarray*}

The likelihood of $(\vartheta ,{\sigma }_{i}^{2},{\delta }_{i})$ is

(27) \begin{eqnarray*}L(\vartheta ,{\sigma }_{i}^{2},{\delta }_{i}{|}{w}_{ij})=\prod _{i=1}^{k} \left( {{n}_{i}\atop {n}_{i(0)}} \right) {\delta }_{i}(1-{\delta }_{i}) \frac{1}{(2\pi {\sigma }_{i}^{2})^{{n}_{i(1)}/2}} \exp \nolimits \left\{ - \frac{1}{2{\sigma }_{i}^{2}} \sum _{j=1}^{{n}_{i(1)}}{ \left( \ln \nolimits {w}_{ij}-\ln \nolimits \left( \frac{\vartheta }{1-{\delta }_{i}} \right) + \frac{{\sigma }_{i}^{2}}{2} \right) }^{2} \right\} \end{eqnarray*}

which enables obtaining the maximum likelihood estimates of ln*ϑ* and ${\sigma }_{i}^{2}$ as

(28) \begin{eqnarray*}\ln \nolimits {\hat {\vartheta }}_{mle} & = \frac{\sum _{i=1}^{k}{\hat {w}}_{mle,i} \left[ {\hat {\mu }}_{i}+\ln \nolimits (1-{\hat {\delta }}_{i}) \right] +N/2}{\sum _{i=1}^{k}{\hat {w}}_{mle,i}} {\hat {\sigma }}_{mle,i}^{2} & =-2+2\sqrt{1+{\hat {\sigma }}_{i}^{2}+{\{\hat {\mu }-\ln \nolimits [\hat {\vartheta }/(1-{\hat {\delta }}_{i})]\}}^{2}}\end{eqnarray*}

where ${\hat {w}}_{mle,i}={n}_{i(1)}/{\hat {\sigma }}_{mle,i}^{2}$ and $\ln {\hat {\vartheta }}_{=} \frac{{\mathop{\sum }\nolimits }_{i=1}^{k}{\hat {w}}_{i} \left[ {\hat {\mu }}_{i}+\ln (1-{\hat {\delta }}_{i}) \right] +N/2}{{\mathop{\sum }\nolimits }_{i=1}^{k}{\hat {w}}_{i}} $; ${\hat {w}}_{i}={n}_{i(1)}/{\hat {\sigma }}_{i}^{2}$. If *δ*_*i*_ = 0, then it becomes the common lognormal mean (see Krishnamoorthy & Oral (2015) for a detailed explanation). By applying central limit theorem, we obtain $ \left( \ln {\hat {\vartheta }}_{mle}-\ln \vartheta \right) \sqrt{{\mathop{\sum }\nolimits }_{i=1}^{k}{\hat {w}}_{mle,i}}\sim N(0,1)$ such that $T={ \left( \ln {\hat {\vartheta }}_{mle}-\ln \vartheta \right) }^{2}{\mathop{\sum }\nolimits }_{i=1}^{k}{\hat {w}}_{mle,i}\sim {\chi }_{{n}_{i(1)}-1}^{2}$. It is well-known that ${\hat {\mu }}_{i}$, ${\hat {\sigma }}_{i}^{2}$ and ${\hat {\delta }}_{i}$ are independent random variables for which ${\hat {\mu }}_{i}\sim N(\ln \left( \frac{\vartheta }{1-{\delta }_{i}} \right) - \frac{{\sigma }_{i}^{2}}{2} ,{\sigma }_{i}^{2}/{n}_{i(1)})$, $({n}_{i(1)}-1){\hat {\sigma }}_{i}^{2}/{\sigma }_{i}^{2}\sim {\chi }_{{n}_{i(1)}-1}^{2}$ and ${\hat {\delta }}_{i}\sim N(\delta ,\delta (1-\delta )/{n}_{i})$ are obtained, respectively. Let $\eta ={\mu }_{i}+{\sigma }_{i}^{2}/2$ so that we can write $T= \frac{{\mathop{\sum }\nolimits }_{i=1}^{k}{\hat {w}}_{mle,i} \left[ {\hat {\mu }}_{i}+\ln (1-{\hat {\delta }}_{i})-\eta -\ln (1-{\delta }_{i}) \right] +N/2}{{\mathop{\sum }\nolimits }_{i=1}^{k}{\hat {w}}_{mle,i}} $. It can be seen that the distribution of *T* is complicated, possibly depending on nuisance parameters ${\sigma }_{i}^{2}$ and *δ*_*i*_, but not on ln*ϑ*. Thus, the exact distribution of T is unknown in practice, and so we propose the PB pivotal variable corresponding to *T*^*PB*^ as

(29) \begin{eqnarray*}{T}^{PB}={ \left( \ln \nolimits {\hat {\vartheta }}_{mle}^{PB}-\ln \nolimits \hat {\vartheta } \right) }^{2}\sum _{i=1}^{k}{\hat {w}}_{mle,i}^{PB}\end{eqnarray*}

where $\ln {\hat {\vartheta }}_{mle}^{PB}= \frac{{\mathop{\sum }\nolimits }_{i=1}^{k}{\hat {w}}_{mle,i}^{PB} \left[ {\hat {\mu }}_{i}^{PB}+\ln (1-{\hat {\delta }}_{i}^{PB}) \right] +N/2}{{\mathop{\sum }\nolimits }_{i=1}^{k}{\hat {w}}_{mle,i}^{PB}} $, ${\hat {w}}_{i}^{PB}={n}_{i(1)}/{\hat {\sigma }}_{i}^{2B}$, ${\hat {\mu }}_{i}^{PB}\sim N({\hat {\mu }}_{i}^{B},{\hat {\sigma }}_{i}^{2B}/{n}_{i(1)})$, ${\hat {\sigma }}_{i}^{2PB}\sim {\hat {\sigma }}_{i}^{B2}{\chi }_{{n}_{i(1)}-1}^{2}/({n}_{i(1)}-1)$ and ${\hat {\delta }}^{PB}\sim beta({n}_{i(0)}^{B}+0.5,{n}_{i(1)}^{B}+0.5)$, ${n}_{i(0)}^{B}={n}_{i}{\hat {\delta }}_{i}^{B}$, and ${n}_{i(1)}^{B}={n}_{i}-{n}_{i(0)}^{B}$. Note that ${\hat {\mu }}_{i}^{B}$, ${\hat {\sigma }}_{i}^{2B}$, and ${\hat {\delta }}_{i}^{B}$ are the observed values of ${\hat {\mu }}_{i}$, ${\hat {\sigma }}_{i}^{2}$, and ${\hat {\delta }}_{i}$, respectively, from random sampling with replacement based on the bootstrap approach. Thus, the 100(1 − *ζ*)% PB interval for *ϑ* is given by

(30) \begin{eqnarray*}C{I}_{\vartheta }^{(pb)}=\exp \nolimits \left[ \ln \nolimits {\hat {\vartheta }}_{mle}\mp \sqrt{{q}_{\zeta }^{PB}/\sum _{i=1}^{k}{\hat {w}}_{mle,i}} \right] \end{eqnarray*}

where ${q}_{\zeta }^{PB}$ denotes the (1 − *ζ*)^th^ percentile of distribution of *T*^*PB*^. The PB interval can be constructed as shown in ‘Algorithm 4’.

#### Algorithm 4: PB

(1) Compute ${\hat {\mu }}_{i}$, ${\hat {\sigma }}_{i}^{2}$ and $\hat {\delta }$ leading to obtain $\ln \hat {\vartheta }$.
(2) Compute $\ln {\hat {\vartheta }}_{mle}$ and ${\hat {\sigma }}_{mle,i}^{2}$.
(3) Generate ${\hat {\mu }}_{i}^{PB}$, ${\hat {\sigma }}_{i}^{2PB}$ and ${\hat {\delta }}_{i}^{PB}$ leading to compute $\ln {\hat {\vartheta }}_{mle}^{PB}$.
(4) Repeat steps 1-3, a number of time *m* = 2500, compute *T*^*PB*^ to obtain ${q}_{\zeta }^{PB}$.
(5) Compute 95%PB interval for *ϑ*, as given in Eq. (30).

### Highest posterior density intervals

The HPD interval is constructed from the posterior distribution, as defined by Box & Tiao (1973). Note that the prior of *ϑ*_*i*_ is updated with its likelihood function thereby obtaining the posterior distribution based on the Bayesian approach. Recall that ${W}_{ij}\sim \Delta ({\mu }_{i},{\sigma }_{i}^{2},{\delta }_{i})$, then the likelihood is given by

(31) \begin{eqnarray*}P({w}_{ij}{|}{\mu }_{i},{\sigma }_{i}^{2},{\delta }_{i})\propto \prod _{i=1}^{k}{\delta }_{i}^{{n}_{i(0)}}(1-{\delta }_{i})^{{n}_{i(1)}}({\sigma }_{i}^{2})^{-{n}_{i(1)}/2}\exp \nolimits \left\{ - \frac{1}{2{\sigma }_{i}^{2}} \sum _{j=1}^{{n}_{i(1)}}{ \left( \ln \nolimits {w}_{ij}-{\mu }_{i} \right) }^{2} \right\} .\end{eqnarray*}

For *k* individual samples, Miroshnikov, Wei & Conlon (2015) described the pooled independent sub-posterior samples toward the joint posterior distributions *ϑ* are combined using weighted averages as follows:

(32) \begin{eqnarray*}{\vartheta }^{post}=\sum _{i=1}^{k}{w}_{i}{\vartheta }_{i}^{post}{ \left( \sum _{i=1}^{k}{w}_{i} \right) }^{-1}\end{eqnarray*}

where ${\vartheta }_{i}^{post}$ are the posterior samples of *ϑ*_*i*_, for *i* = 1, 2, …, *k*. The inverse of the sample variance is used to weight the posterior based on the *i*th samples is denoted as ${w}_{i}=Va{r}^{-1}({\hat {\vartheta }}_{i}{|}{w}_{ij})$. Different priors have been developed for estimating the common delta-lognormal mean, two of which are derived in the following subsections.

#### Jeffreys’ rule prior

Harvey & van der Merwe (2012) defined this prior as

(33) \begin{eqnarray*}P(\vartheta )_{JR}\propto \prod _{i=1}^{k}{\sigma }_{i}^{-3}{\delta }_{i}^{-1/2}(1-{\delta }_{i})^{1/2}\end{eqnarray*}

which is combined with the likelihood Eq. (34) to obtain the posterior of *ϑ* as

(34) \begin{eqnarray*}P({w}_{ij}{|}\vartheta )& \propto & \prod _{i=1}^{k}{\delta }_{i}^{{n}_{i(0)}-1/2}(1-{\delta }_{i})^{{n}_{i(1)}+1/2}({\sigma }_{i}^{2})^{-({n}_{i(1)}+3)/2}\exp \nolimits \left\{ - \frac{1}{2{\sigma }_{i}^{2}} \sum _{j=1}^{{n}_{i(1)}}{ \left( \ln \nolimits {w}_{ij}-{\mu }_{i} \right) }^{2} \right\} \nonumber\\\displaystyle & \propto & \prod _{i=1}^{k}{\delta }_{i}^{({n}_{i(0)}+1/2)-1}(1-{\delta }_{i})^{({n}_{i(1)}+3/2)-1}({\sigma }_{i}^{2})^{- \frac{({n}_{i(1)}+1)}{2} -1}\nonumber\\\displaystyle & & \exp \nolimits \left\{ - \frac{1}{2{\sigma }_{i}^{2}} \left[ ({n}_{i(1)}-1){\hat {\sigma }}_{i}^{2}+{n}_{i(1)}({\hat {\mu }}_{i}-{\mu }_{i})^{2} \right] \right\} .\end{eqnarray*}

This leads to obtaining the marginal posterior distributions of *μ*_*i*_, ${\sigma }_{i}^{2}$ and *δ*_*i*_ as

(35) \begin{eqnarray*}{\mu }_{i}^{(JR)}{|}{\sigma }_{i,JR}^{2},{w}_{ij}\sim N \left( {\hat {\mu }}_{i},{\sigma }_{i}^{2(JR)}/{n}_{i(1)} \right) {\sigma }_{i}^{2(JR)}{|}{w}_{ij}\sim IG \left( ({n}_{i(1)}+1)/2,({n}_{i(1)}+1){\hat {\sigma }}_{i}^{2}/2 \right) {\delta }_{i}^{(JR)}{|}{w}_{ij}\sim beta \left( {n}_{i(0)}+1/2,{n}_{i(1)}+3/2 \right) .\end{eqnarray*}

The pooled posterior of *ϑ* is weighted by its inversely estimated variance as follows:

(36) \begin{eqnarray*}{\vartheta }^{post}=\sum _{i=1}^{k}{w}_{i}^{(JR)}{\vartheta }_{i}^{(JR)_{p}}{ \left( \sum _{i=1}^{k}{w}_{i}^{(JR)} \right) }^{-1}\end{eqnarray*}

where

${\vartheta }_{i}^{(JR)_{p}}=(1-{\delta }_{i}^{(JR)})\exp ({\mu }_{i}^{(JR)}+{\sigma }_{i}^{2(JR)}/2)$

${w}_{i}^{(JR)}={ \left\{ {n}_{i}^{-1}\exp (2{\mu }_{i}^{(JR)}+{\sigma }_{i}^{2(JR)}) \left[ {\delta }_{i}^{(JR)}(1-{\delta }_{i}^{(JR)})+ \frac{1}{2} (1-{\delta }_{i}^{(JR)})(2{\sigma }_{i}^{2(JR)}+{\sigma }_{i}^{4(JR)}) \right] \right\} }^{-1}$.

From Eq. (36), the 100(1 − *ζ*)%HPD-based Jeffreys’ rule prior (HPD-JR) for *ϑ* is constructed as follows:

#### Normal-gamma-beta prior

Maneerat, Niwitpong & Niwitpong (2020) proposed a HPD based on the normal-gamma prior for the ratio of delta-lognormal variances that worked better than the HPD-JR of Harvey & van der Merwe (2012). Suppose that ***Y*** = ln***W*** be a random variable of normal distribution with mean *μ* = (*μ*_1_, *μ*_2_, …, *μ*_*k*_) and precision *λ* = (*λ*_1_, *λ*_2_, …, *λ*_*k*_) where ***W*** ∼ *LN*(*μ*, *λ*) and ${\lambda }_{i}={\sigma }_{i}^{-2}$. The HPD-based normal-gamma-beta prior (HPD-NGB) of *ϑ* = (*μ*_*i*_, *λ*_*i*_, *δ*_*i*_)′ is defined as

(37) \begin{eqnarray*}P(\vartheta )\propto \prod _{i=1}^{k}{\lambda }_{i}^{-1}[{\delta }_{i}(1-{\delta }_{i})]^{-1/2}\end{eqnarray*}

where (*μ*_*i*_, *λ*_*i*_) follows a normal-gamma distribution, and *δ*_*i*_ follows a beta distribution, denoted as (*μ*_*i*_, *λ*_*i*_) ∼ *NG*(*μ*_*i*_, *λ*_*i*_|*μ*, *k*_*i*(0)_ = 0, *α*_*i*(0)_ =  − 1∕2, *β*_*i*(0)_ = 0) and *δ*_*i*_ ∼ *beta*(1∕2, 1∕2), respectively. When the the prior Eq. (37) is combined with the likelihood Eq. (34), then the posterior density of *ϑ* becomes

(38) \begin{eqnarray*}P(\vartheta {|}{w}_{ij})& \propto & \prod _{i=1}^{k}{\delta }_{i}^{{n}_{i(0)}-1/2}(1-{\delta }_{i})^{{n}_{i(1)}-1/2}{\lambda }_{i}^{ \frac{{n}_{i(1)}-1}{2} -1}\exp \nolimits \left\{ - \frac{{\lambda }_{i}}{2} \sum _{j=1}^{{n}_{i(1)}}(\ln \nolimits {w}_{ij}-{\hat {\mu }}_{i})^{2} \right\} {\lambda }_{i}^{1/2}\nonumber\\\displaystyle & & \exp \nolimits \left\{ - \frac{{n}_{i(1)}{\lambda }_{i}}{2} ({\mu }_{i}-{\mu }_{i}^{\ast })^{2} \right\} \end{eqnarray*}

which can be integrated out to obtain the marginal posterior distributions of *μ*_*i*_, *λ*_*i*_ and *δ*_*i*_ as follows:

(39) \begin{eqnarray*}{\mu }_{i}^{(NGB)}{|}{w}_{ij}\sim {t}_{df} \left( {\mu }_{i}{|}{\hat {\mu }}_{i},\sum _{j=1}^{{n}_{i(1)}}(\ln \nolimits {w}_{ij}-{\hat {\mu }}_{i})^{2}/[{n}_{i(1)}({n}_{i(1)}-1)] \right) {\lambda }_{i}^{(NGB)}{|}{w}_{ij}\sim G \left( {\lambda }_{i}{|}({n}_{i(1)}-1)/2,\sum _{j=1}^{{n}_{i(1)}}(\ln \nolimits {w}_{ij}-{\hat {\mu }}_{i})^{2}/2 \right) {\delta }_{i}^{(NGB)}{|}{w}_{ij}\sim beta \left( {n}_{i(0)}+1/2,{n}_{i(1)}+1/2 \right) \end{eqnarray*}

where *df* = 2 (*n*_*i*(1)_ − 1) and ${\sigma }_{i}^{2(NGB)}{|}{w}_{ij}\sim IG \left( {\sigma }_{i}^{2}{|}({n}_{i(1)}-1)/2,{\mathop{\sum }\nolimits }_{j=1}^{{n}_{i(1)}}(\ln {w}_{ij}-{\hat {\mu }}_{i})^{2}/2 \right) $. Similarly, the pooled posterior of *ϑ* is given by

(40) \begin{eqnarray*}{\vartheta }^{post}=\sum _{i=1}^{k}{w}_{i}^{(NGB)}{\vartheta }_{i}^{(NGB)_{p}}{ \left( \sum _{i=1}^{k}{w}_{i}^{(NGB)} \right) }^{-1}\end{eqnarray*}

where ${\vartheta }_{i}^{(NGB)_{p}}=(1-{\delta }_{i}^{(NGB)})\exp ({\mu }_{i}^{(NGB)}+{\sigma }_{i}^{2(NGB)}/2){w}_{i}^{(NGB)}$ $={ \left\{ {n}_{i}^{-1}\exp (2{\mu }_{i}^{(NGB)}+{\sigma }_{i}^{2(NGB)}) \left[ {\delta }_{i}^{(NGB)}(1-{\delta }_{i}^{(NGB)}) \frac{1}{2} (1-{\delta }_{i}^{(NGB)})(2{\sigma }_{i}^{2(NGB)}+{\sigma }_{i}^{4(NGB)}) \right] \right\} }^{-1}$.

Hence, the 100(1 − *ζ*)%HPD-HGB for *ϑ* is constructed in Eq. (40). ‘Algorithm 5’ details the steps to construct the HPD-JR and HPD-NGB.

#### Algorithm 5: HPD-JR and HPD-NGB

(1) Compute ${\hat {\mu }}_{i}$, ${\hat {\sigma }}_{i}^{2}$ and $\hat {\delta }$.
(2) Generate the posterior densities of *μ*_*i*_, ${\sigma }_{i}^{2}$ and *δ*_*i*_ based-Jeffreys’ rule (JR) and normal-gamma-beta (NGB) priors, as given in Eq. (35) and Eq. (39), respectively.
(3) Compute the pooled posterior of *ϑ* based on JR and NGB priors, as given in Eq. (36) and Eq. (40), respectively.
(4) Compute 95%HPD-JR and HPD-NGB for *ϑ*, defined by Box & Tiao (1973).

## Simulation Studies and Results

The performances of the CIs were assessed by comparing their coverage probabilities (CPs) and average length (ALs) using Monte Carlo simulation. The best-performing CI is the one where the CP is closest to or greater than the nominal confidence level 1 − *ζ* while also having an AL with the narrowest width. The CIs for the common delta-lognormal mean constructed using FGCI, LS, MOVER, PB, HPD-JR, and HPD-NGB were assessed in the study, the parameter settings for which are provided in Table 1. The number of generated random samples was fixed at *M* = 5000. For FGCI, the number of FGPQs was *Q* = 2500 for each set of 5,000 random samples. ‘Algorithm 6’ shows the computational steps to estimate the CP and AL performances of all of the methods.

**Table 1:** Parameter settings for sample cases *k* = 2, 5, 10.

Scenarios	(*n*_1_, …, *n*_*k*_)	(*δ*_1_, …, *δ*_*k*_)	$({\sigma }_{1}^{2},\ldots ,{\sigma }_{k}^{2})$
*k* = 2			
1–9	(30_2_)	(0.1,0.2), (0.2,0.5), (0.3,0.7)	(1,2), (2,4), (3,5)
10–18	(30,50)	(0.1,0.2), (0.2,0.5), (0.3,0.7)	(1,2), (2,4), (3,5)
19–27	(50_2_)	(0.1,0.2), (0.2,0.5), (0.3,0.7)	(1,2), (2,4), (3,5)
28–36	(50,100)	(0.1,0.2), (0.2,0.5), (0.3,0.7)	(1,2), (2,4), (3,5)
37–45	(100_2_)	(0.1,0.2), (0.2,0.5), (0.3,0.7)	(1,2), (2,4), (3,5)
*k* = 5			
46–54	(30_5_)	(0.05, 0.1_2_, 0.2_2_), (0.2_2_, 0.4_3_), (0.5_2_, 0.7_3_)	(1_2_, 2_3_), (2_2_, 3_3_), (3_2_, 5_3_)
55–63	(30_2_, 50_3_)	(0.05, 0.1_2_, 0.2_2_), (0.2_2_, 0.4_3_), (0.5_2_, 0.7_3_)	(1_2_, 2_3_), (2_2_, 3_3_), (3_2_, 5_3_)
64–72	(30_2_, 50_2_, 100)	(0.05, 0.1_2_, 0.2_2_), (0.2_2_, 0.4_3_), (0.5_2_, 0.7_3_)	(1_2_, 2_3_), (2_2_, 3_3_), (3_2_, 5_3_)
73–81	(30, 50_2_, 100_2_)	(0.05, 0.1_2_, 0.2_2_), (0.2_2_, 0.4_3_), (0.5_2_, 0.7_3_)	(1_2_, 2_3_), (2_2_, 3_3_), (3_2_, 5_3_)
82–90	(50_5_)	(0.05, 0.1_2_, 0.2_2_), (0.2_2_, 0.4_3_), (0.5_2_, 0.7_3_)	(1_2_, 2_3_), (2_2_, 3_3_), (3_2_, 5_3_)
91–99	(50_2_, 100_3_)	(0.05, 0.1_2_, 0.2_2_), (0.2_2_, 0.4_3_), (0.5_2_, 0.7_3_)	(1_2_, 2_3_), (2_2_, 3_3_), (3_2_, 5_3_)
100–108	(100_5_)	(0.05, 0.1_2_, 0.2_2_), (0.2_2_, 0.4_3_), (0.5_2_, 0.7_3_)	(1_2_, 2_3_), (2_2_, 3_3_), (3_2_, 5_3_)
*k* = 10			
109–114	(30_5_, 50_5_)	(0.1_5_, 0.2_5_), (0.2_5_, 0.5_5_)	(1_5_, 2_5_), (2_5_, 4_5_), (3_5_, 5_5_)
115–120	(30_3_, 50_3_, 100_4_)	(0.1_5_, 0.2_5_), (0.2_5_, 0.5_5_)	(1_5_, 2_5_), (2_5_, 4_5_), (3_5_, 5_5_)
121–126	(50_5_, 100_5_)	(0.1_5_, 0.2_5_), (0.2_5_, 0.5_5_)	(1_5_, 2_5_), (2_5_, 4_5_), (3_5_, 5_5_)

**Notes.**

Note: (30_5_) stands for (30, 30, 30, 30, 30).

**Table 2:** Performance measures of 95%CIs for *ϑ*: 2 sample cases.

Scenarios	CP						AL					
	FG	LS	MO	PB	HJ	HN	FG	LS	MO	PB	HJ	HN
*k* = 2												
1	**0.959**	0.897	0.967	0.994	0.916	0.941	**1.556**	1.296	2.005	2.324	1.353	1.436
2	**0.958**	0.857	0.947	0.996	0.924	0.941	**5.169**	3.770	7.287	8.631	4.186	4.335
3	**0.963**	0.821	0.959	0.996	0.919	0.932	**13.088**	8.675	23.312	22.883	9.905	10.220
4	**0.962**	0.886	0.978	0.995	0.917	0.939	**1.487**	1.211	2.181	2.155	1.247	1.386
5	**0.953**	0.832	0.962	0.995	0.913	0.922	**4.875**	3.487	9.881	7.818	3.811	4.066
6	**0.951**	0.793	0.971	0.991	0.901	0.912	**12.311**	7.740	37.615	21.129	8.875	9.378
7	**0.961**	0.829	0.972	0.982	0.920	0.940	**1.511**	1.095	3.968	2.173	1.224	1.406
8	**0.950**	0.778	0.974	0.995	0.900	0.911	**4.821**	3.123	293.620	7.649	3.566	3.916
9	0.939	0.725	0.973	**0.988**	0.866	0.887	13.159	7.067	8.0e4	**23.632**	8.680	9.419
10	**0.960**	0.900	0.965	0.992	0.915	0.941	**1.503**	1.249	1.936	2.225	1.362	1.395
11	**0.961**	0.848	0.941	0.992	0.924	0.940	**5.128**	3.712	6.765	8.667	4.298	4.368
12	**0.965**	0.819	0.952	0.998	0.919	0.931	**12.297**	8.382	20.057	21.597	9.819	9.894
13	**0.960**	0.896	0.977	0.992	0.917	0.942	**1.366**	1.147	1.909	2.004	1.203	1.271
14	**0.961**	0.851	0.964	0.996	0.916	0.931	**4.593**	3.422	7.236	7.458	3.761	3.889
15	**0.949**	0.790	0.958	0.994	0.894	0.905	**11.116**	7.517	22.293	19.310	8.507	8.718
16	**0.963**	0.860	0.972	0.974	0.928	0.943	**1.354**	1.033	2.141	1.928	1.155	1.257
17	**0.952**	0.803	0.976	0.992	0.900	0.917	**4.397**	3.048	10.772	6.889	3.418	3.630
18	0.940	0.737	**0.968**	0.989	0.872	0.889	11.065	6.663	**43.755**	19.011	7.903	8.247
19	**0.961**	0.914	0.966	0.992	0.921	0.946	**1.153**	1.009	1.382	1.696	1.043	1.076
20	**0.965**	0.895	0.946	0.991	0.938	0.949	**3.668**	2.924	4.309	5.981	3.178	3.229
21	**0.962**	0.863	0.952	0.996	0.930	0.940	**8.747**	6.665	11.805	14.651	7.272	7.395
22	**0.958**	0.910	0.978	0.985	0.919	0.944	**1.091**	0.945	1.414	1.555	0.945	1.031
23	**0.965**	0.883	0.969	0.996	0.926	0.937	**3.336**	2.695	4.578	5.204	2.811	2.950
24	**0.961**	0.840	0.972	0.995	0.921	0.928	**7.887**	5.987	13.164	12.757	6.338	6.605
25	**0.969**	0.868	0.980	0.958	0.930	0.953	**1.120**	0.866	1.610	1.503	0.937	1.070
26	**0.954**	0.839	0.970	0.997	0.916	0.926	**3.208**	2.433	6.544	4.735	2.621	2.830
27	**0.946**	0.773	0.970	0.992	0.893	0.903	**7.803**	5.443	26.105	12.382	6.011	6.376
28	**0.958**	0.912	0.972	0.979	0.916	0.947	**1.119**	0.952	1.397	1.615	1.054	1.051
29	**0.956**	0.872	0.921	0.958	0.927	0.943	**3.745**	2.836	4.238	6.098	3.338	3.330
30	**0.961**	0.846	0.937	0.987	0.925	0.936	**8.488**	6.274	10.833	13.991	7.332	7.320
31	**0.962**	0.927	0.985	0.978	0.919	0.949	**0.984**	0.876	1.322	1.433	0.908	0.929
32	**0.960**	0.880	0.958	0.992	0.925	0.940	**3.214**	2.618	4.169	5.150	2.818	2.860
33	**0.958**	0.838	0.960	0.994	0.910	0.925	**7.360**	5.744	10.824	12.105	6.256	6.279
34	**0.963**	0.888	0.977	0.922	0.938	0.954	**0.975**	0.785	1.322	1.352	0.876	0.922
35	**0.958**	0.860	0.971	0.995	0.917	0.929	**2.915**	2.343	4.321	4.424	2.486	2.586
36	**0.951**	0.820	0.973	0.995	0.901	0.916	**6.726**	5.103	11.951	10.823	5.511	5.626
37	**0.957**	0.935	0.960	0.970	0.927	0.948	**0.802**	0.722	0.923	1.168	0.743	0.753
38	**0.955**	0.916	0.926	0.953	0.942	0.948	**2.442**	2.044	2.541	3.935	2.220	2.219
39	**0.957**	0.888	0.939	0.981	0.937	0.945	**5.608**	4.594	6.295	9.049	4.984	4.998
40	**0.961**	0.942	0.975	0.957	0.924	0.954	**0.740**	0.679	0.911	1.062	0.659	0.702
41	**0.961**	0.920	0.960	0.988	0.933	0.950	**2.199**	1.925	2.558	3.401	1.958	2.012
42	**0.955**	0.875	0.960	0.994	0.925	0.931	**4.976**	4.209	6.298	7.813	4.318	4.439
43	**0.967**	0.909	0.980	0.863	0.937	0.960	**0.773**	0.625	0.972	1.012	0.659	0.743
44	**0.960**	0.896	0.970	0.993	0.928	0.939	**2.076**	1.750	2.684	3.013	1.788	1.921
45	**0.952**	0.835	0.970	0.996	0.908	0.914	**4.683**	3.786	7.007	7.008	3.952	4.182

**Notes.**

FG fiducial generalized confidence interval MO method of variance estimates HJ HPD-based Jeffreys’ rule prior HPD-JR HN, HPD-based normal-gamma-beta prior

Bold denoted as the best-performing method each case.

**Figure 1:**
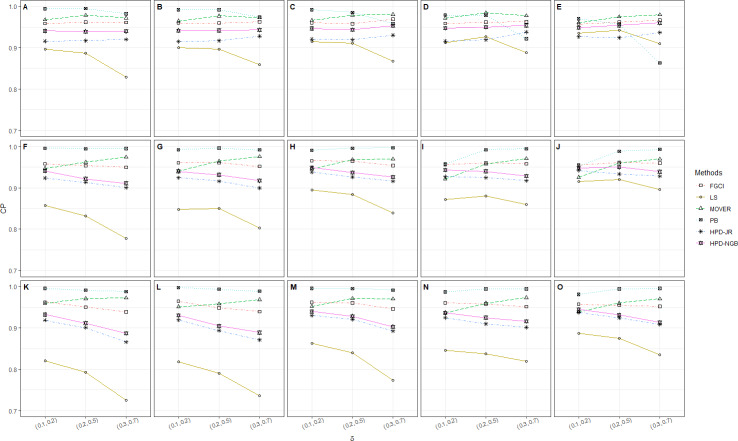
CP performances of 95%CIs for *ϑ*: 2 sample cases in the following cases (sample sizes, variances): (A) (30_2_, 1, 2), (B) (30, 30, 2, 4), (C) (30, 30, 3, 5), (D) (30, 50, 1, 2), (E) (30, 50, 2, 4), (F) (30, 50, 3, 5), (G) (50_2_, 1, 2), (H) (50_2_, 2, 4), (I) (50_2_, 3, 5), (J) (50, 100, 1, 2), (K) (50, 100, 2, 4), (L) (50, 100, 3, 5), (M) (100_2_, 1, 2), (N) (100_2_, 2, 4), (O) (100_2_, 3, 5).

### Algorithm 6: Comparison of CPs and ALs for all CIs

(1) For *g* = 1 to *M*. Generate ${w}_{ij}\sim \Delta ({\mu }_{i},{\sigma }_{i}^{2},{\delta }_{i})$.
(2) Compute the unbiased estimates ${\hat {\mu }}_{i}$, ${\hat {\sigma }}_{i}^{2}$ and $\hat {\delta }$.
(3) Compute the 95%CIs for *ϑ* based on FGCI, LS, MOVER, PB and the HPDs via Algorithm 1, 2, 3, 4 and 5, respectively.
(4) Let *A*_*g*_ = 1 if *ϑ* falls within the intervals of FGCI, LS, MOVER, PB or the HPDs, else *A*_*g*_ = 0.
(5) The CP and AL for each method are obtained by $\text{CP}=(1/M){\mathop{\sum }\nolimits }_{g=1}^{M}{A}_{g}$ and AL = (*U* − *L*)∕*M*, respectively, where *U* and *L* are the upper and lower confidence limits, respectively. (end *g* loop)

**Figure 2:**
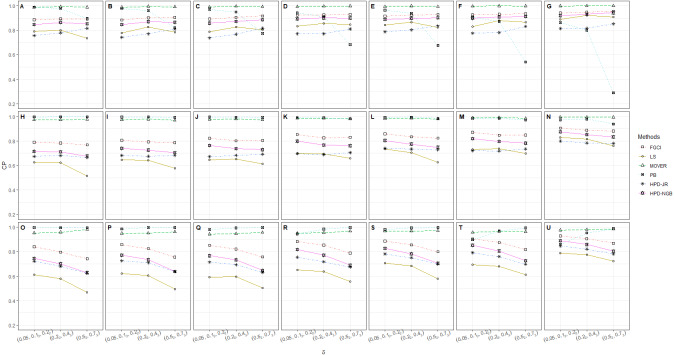
CP performances of 95%CIs for *ϑ*: 5 sample cases in the following cases (sample sizes, variances): (A) (30_5_, 1_2_, 2_3_), (B) (30_5_, 2_2_, 3_3_), (C) (30_5_, 3_2_, 5_3_), (D) (30_2_, 50_3_, 1_2_, 2_3_), (E) (30_2_, 50_3_, 2_2_, 3_3_), (F) (30_2_, 50_3_, 3_2_, 5_3_), (G) (30_2_, 50_3_, 100, 1_2_, 2_3_), (H) (30_2_, 50_3_, 100, 2_2_, 3_3_), (I) (30_2_, 50_3_, 100, 3_2_, 5_3_), (J) (30, 50_2_, 100_2_, 1_2_, 2_3_), (K) (30, 50_2_, 100_2_, 1_2_, 2_3_), (L) (30, 50_2_, 100_2_, 1_2_, 2_3_), (M) (50_5_, 1_2_, 2_3_), (N) (50_5_, 2_2_, 3_3_), (O) (50_5_, 3_2_, 5_3_), (P) (50_2_, 100_3_, 1_2_, 2_3_), (Q) (50_2_, 100_3_, 2_2_, 3_3_), (R) (50_2_, 100_3_, 3_2_, 5_3_), (S) (100_5_, 1_2_, 2_3_), (T) (100_5_, 2_2_, 3_3_), (U) (100_5_, 3_2_, 5_3_).

**Figure 3:**
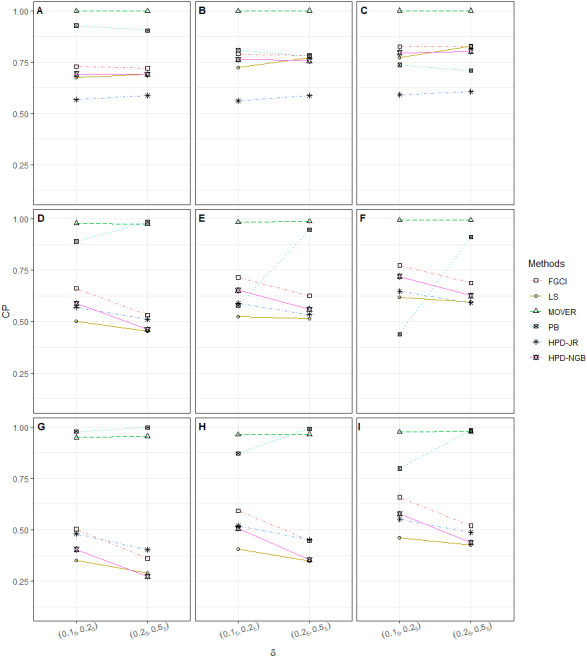
CP performances of 95%CIs for *ϑ*: 10 sample cases in the following cases (sample sizes, variances): (A) (30_5_, 50_5_, 1_2_, 2_5_), (B) (30_5_, 50_5_, 2_5_, 4_5_), (C) (30_5_, 50_5_, 3_5_, 5_5_), (D) (30_3_, 50_3_, 100_4_, 1_2_, 2_5_), (E) (30_3_, 50_3_, 100_4_, 2_5_, 4_5_), (F) (30_3_, 50_3_, 100_4_, 3_5_, 5_5_), (G) (50_5_, 100_5_, 1_5_, 2_5_), (H) (50_5_, 100_5_, 2_5_, 4_5_), (I) (50_5_, 100_5_, 3_2_, 5_3_).

The numerical results for the CI performances are presented in terms of CP and AL for various sample cases. For *k* = 2 (Table 2 and Fig. 1), FGCI performed well for small-to-moderate sample sizes, as well as for large ${\sigma }_{i}^{2}$ and a moderate-to-large sample size. HPD-NGB attained stable and the best CP and AL values for small ${\sigma }_{i}^{2}$ and a moderate-to-large sample size. MOVER and PB attained correct CPs but wider ALs than the other methods whereas LS and HPD-JR had lower CPs and narrower ALs. For *k* = 5 (Table 3 and Fig. 2), there were only two methods producing better CPs than the other methods in the various situations: MOVER (small *δ*_*i*_ and ${\sigma }_{i}^{2}$) and PB (large *δ*_*i*_ and ${\sigma }_{i}^{2}$). Moreover, the results were similar for *k* = 10 (Table 4 and Fig. 3).

**Table 3:** Performance measures of 95% CIs for *ϑ*: 5 sample cases.

Scenarios	CP						AL					
	FG	LS	MO	PB	HJ	HN	FG	LS	MO	PB	HJ	HN
*k* = 5												
46	0.885	0.790	0.988	**0.989**	0.757	0.846	0.963	0.819	1.794	**1.532**	0.848	0.956
47	0.789	0.627	0.973	**0.996**	0.674	0.715	2.240	1.908	4.982	**3.897**	1.991	2.176
48	0.840	0.613	0.953	**0.997**	0.723	0.746	5.325	4.529	13.769	**12.250**	4.744	4.870
49	0.894	0.800	0.993	**0.978**	0.779	0.864	0.900	0.765	1.825	**1.439**	0.773	0.905
50	0.783	0.623	0.972	**0.998**	0.680	0.711	2.008	1.711	5.203	**3.608**	1.750	1.955
51	0.797	0.580	0.959	**0.996**	0.680	0.701	4.700	4.066	16.626	**11.353**	4.118	4.287
52	0.893	0.735	**0.989**	0.896	0.816	0.853	0.753	0.589	**2.849**	1.433	0.636	0.764
53	0.768	0.517	0.977	**0.997**	0.666	0.676	1.474	1.168	19.967	**3.364**	1.282	1.406
54	0.742	0.467	0.983	**0.996**	0.624	0.629	3.250	2.654	1.5e4	**11.238**	2.817	2.855
55	0.884	0.779	0.988	**0.979**	0.743	0.846	0.940	0.777	1.739	**1.434**	0.857	0.930
56	0.806	0.645	0.973	**0.995**	0.681	0.740	2.204	1.822	4.586	**3.561**	2.045	2.141
57	0.858	0.622	0.949	**0.986**	0.725	0.771	5.620	4.542	12.575	**12.073**	5.122	5.162
58	0.901	0.827	0.995	**0.962**	0.770	0.870	0.845	0.728	1.699	**1.326**	0.771	0.841
59	0.793	0.644	0.978	**0.997**	0.675	0.726	1.904	1.629	4.351	**3.262**	1.750	1.850
60	0.825	0.605	0.952	**0.997**	0.710	0.734	4.753	4.058	12.745	**10.793**	4.373	4.353
61	0.905	0.785	**0.992**	0.822	0.809	0.865	0.685	0.564	**1.632**	1.219	0.620	0.686
62	0.786	0.578	0.969	**0.993**	0.683	0.704	1.368	1.142	4.477	**2.775**	1.260	1.309
63	0.755	0.496	0.963	**0.998**	0.639	0.637	3.177	2.714	18.995	**8.911**	2.884	2.822
64	0.892	0.787	0.991	**0.970**	0.737	0.858	0.928	0.751	1.740	**1.364**	0.872	0.919
65	0.822	0.647	0.975	**0.996**	0.673	0.763	2.168	1.738	4.371	**3.326**	2.047	2.114
66	0.852	0.593	0.943	**0.981**	0.715	0.767	5.710	4.413	12.195	**11.422**	5.267	5.278
67	0.905	0.827	0.996	**0.949**	0.768	0.873	0.816	0.697	1.637	**1.256**	0.770	0.811
68	0.801	0.654	0.979	**0.995**	0.683	0.737	1.839	1.549	4.069	**3.016**	1.753	1.797
69	0.821	0.595	0.947	**0.994**	0.693	0.733	4.806	3.976	12.174	**10.326**	4.431	4.432
70	0.917	0.803	**0.994**	0.775	0.817	0.886	0.650	0.539	**1.499**	1.133	0.616	0.650
71	0.804	0.612	0.973	**0.992**	0.692	0.730	1.310	1.094	3.962	**2.543**	1.236	1.262
72	0.756	0.502	0.958	**0.997**	0.631	0.646	3.158	2.695	16.604	**8.356**	2.888	2.835
73	0.924	0.832	**0.994**	0.942	0.772	0.893	0.822	0.673	**1.505**	1.186	0.856	0.808
74	0.853	0.699	0.985	**0.990**	0.696	0.798	1.971	1.589	3.823	**2.899**	2.000	1.923
75	0.883	0.652	0.952	**0.945**	0.755	0.817	5.330	4.072	9.997	**9.911**	5.224	4.974
76	0.924	0.857	**0.997**	0.913	0.771	0.901	0.723	0.626	**1.418**	1.088	0.746	0.715
77	0.826	0.695	0.986	**0.989**	0.689	0.767	1.670	1.406	3.476	**2.610**	1.692	1.632
78	0.854	0.638	0.955	**0.984**	0.718	0.771	4.456	3.628	9.715	**8.788**	4.311	4.160
79	0.930	0.846	**0.998**	0.683	0.811	0.900	0.581	0.486	**1.253**	0.964	0.586	0.580
80	0.830	0.658	0.981	**0.980**	0.705	0.762	1.215	1.019	3.179	**2.168**	1.225	1.181
81	0.788	0.555	0.967	**0.997**	0.675	0.689	2.992	2.554	11.873	**7.026**	2.927	2.738
82	0.915	0.844	0.993	**0.964**	0.788	0.889	0.769	0.662	1.337	**1.158**	0.692	0.753
83	0.858	0.735	0.982	**0.993**	0.741	0.804	1.882	1.599	3.605	**2.920**	1.698	1.825
84	0.886	0.705	0.969	**0.981**	0.782	0.827	4.650	3.895	8.767	**9.068**	4.208	4.335
85	0.925	0.865	**0.998**	0.939	0.803	0.897	0.707	0.618	**1.315**	1.068	0.618	0.700
86	0.834	0.705	0.987	**0.994**	0.735	0.775	1.683	1.439	3.493	**2.683**	1.482	1.642
87	0.855	0.684	0.968	**0.994**	0.751	0.783	4.027	3.489	8.924	**8.068**	3.613	3.766
88	0.929	0.824	**0.994**	0.677	0.835	0.903	0.611	0.495	**1.322**	0.993	0.515	0.616
89	0.823	0.627	0.981	**0.985**	0.729	0.749	1.284	1.045	3.692	**2.296**	1.121	1.250
90	0.799	0.578	0.972	**0.997**	0.699	0.705	2.875	2.453	13.603	**6.644**	2.519	2.641
91	0.927	0.831	**0.997**	0.906	0.777	0.898	0.753	0.614	**1.389**	1.064	0.703	0.735
92	0.871	0.731	0.988	**0.986**	0.720	0.820	1.821	1.466	3.459	**2.601**	1.721	1.769
93	0.905	0.693	0.957	**0.897**	0.791	0.852	5.015	3.768	8.461	**8.829**	4.621	4.690
94	0.931	0.879	**0.999**	0.873	0.781	0.909	0.651	0.571	**1.279**	0.972	0.608	0.639
95	0.847	0.738	0.991	**0.986**	0.719	0.797	1.541	1.313	3.117	**2.351**	1.447	1.499
96	0.875	0.679	0.966	**0.969**	0.760	0.806	4.125	3.374	8.002	**7.707**	3.808	3.865
97	0.935	0.866	**0.998**	0.541	0.832	0.911	0.529	0.450	**1.097**	0.856	0.493	0.523
98	0.848	0.697	0.986	**0.971**	0.735	0.782	1.126	0.956	2.572	**1.916**	1.060	1.091
99	0.817	0.613	0.963	**0.994**	0.698	0.725	2.784	2.418	7.510	**6.042**	2.565	2.571
100	0.941	0.888	**0.998**	0.863	0.813	0.920	0.557	0.484	**0.954**	0.806	0.510	0.536
101	0.906	0.827	0.995	**0.973**	0.799	0.875	1.413	1.201	2.515	**2.029**	1.288	1.361
102	0.929	0.790	**0.975**	0.861	0.845	0.889	3.639	2.946	**5.529**	6.174	3.365	3.428
103	0.948	0.923	**1.000**	0.801	0.816	0.931	0.501	0.456	**0.909**	0.741	0.452	0.487
104	0.888	0.816	0.996	**0.978**	0.784	0.853	1.253	1.095	2.373	**1.852**	1.121	1.216
105	0.905	0.775	0.981	**0.953**	0.822	0.859	3.147	2.678	5.326	**5.441**	2.893	2.975
106	0.955	0.907	**0.999**	0.289	0.852	0.943	0.438	0.372	**0.838**	0.668	0.373	0.433
107	0.881	0.761	**0.994**	0.939	0.781	0.833	0.992	0.823	**2.044**	1.536	0.863	0.972
108	0.868	0.722	0.984	**0.987**	0.781	0.805	2.331	2.005	5.072	**4.308**	2.088	2.208

**Notes.**

FG fiducial generalized confidence interval MO method of variance estimates HJ HPD-based Jeffreys’ rule prior HPD-JR HN, HPD-based normal-gamma-beta prior

Bold denoted as the best-performing method each case.

**Table 4:** Performance measures of 95% CIs for *ϑ*: 10 sample cases.

	CP						AL					
Scenarios	FG	LS	MO	PB	HJ	HN	FG	LS	MO	PB	HJ	HN
*k* = 10												
109	0.728	0.675	**0.998**	0.927	0.566	0.692	0.612	0.501	**1.554**	0.932	0.545	0.623
110	0.661	0.500	**0.979**	0.891	0.570	0.588	1.644	1.291	**3.867**	3.278	1.500	1.637
111	0.504	0.352	0.950	**0.978**	0.481	0.404	3.159	2.561	8.645	**7.286**	2.996	3.076
112	0.720	0.692	**0.999**	0.904	0.587	0.690	0.557	0.459	**1.519**	0.832	0.483	0.574
113	0.532	0.452	0.976	**0.985**	0.512	0.462	1.393	1.159	3.853	**2.682**	1.260	1.404
114	0.361	0.290	0.955	**0.998**	0.403	0.274	2.556	2.218	8.570	**5.943**	2.411	2.505
115	0.789	0.723	**0.999**	0.808	0.561	0.762	0.554	0.440	**1.416**	0.789	0.546	0.560
116	0.716	0.524	**0.985**	0.578	0.590	0.653	1.635	1.180	**3.478**	2.915	1.559	1.624
117	0.593	0.406	**0.964**	0.872	0.519	0.507	3.289	2.406	**7.754**	6.380	3.189	3.214
118	0.782	0.773	**1.000**	0.780	0.586	0.758	0.477	0.404	**1.317**	0.696	0.474	0.483
119	0.626	0.514	0.988	**0.947**	0.535	0.561	1.337	1.076	3.348	**2.360**	1.284	1.341
120	0.447	0.347	0.965	**0.992**	0.450	0.355	2.570	2.108	7.290	**5.180**	2.506	2.531
121	0.826	0.773	**1.000**	0.736	0.592	0.796	0.488	0.399	**1.266**	0.695	0.444	0.486
122	0.774	0.620	**0.994**	0.438	0.647	0.720	1.460	1.086	**3.072**	2.512	1.328	1.438
123	0.659	0.460	**0.977**	0.798	0.553	0.577	3.002	2.236	**6.597**	5.502	2.775	2.921
124	0.828	0.826	**1.000**	0.708	0.606	0.802	0.426	0.368	**1.187**	0.615	0.387	0.427
125	0.688	0.595	**0.995**	0.912	0.591	0.627	1.205	0.992	**2.912**	2.039	1.094	1.197
126	0.520	0.426	0.979	**0.984**	0.486	0.439	2.390	1.989	6.222	**4.479**	2.224	2.344

**Notes.**

FG fiducial generalized confidence interval MO method of variance estimates recovery HJ HPD-based Jeffreys’ rule prior HPD-JR HN, HPD-based normal-gamma-beta prior

Bold denoted as the best-performing method each case.

As previously mentioned, our findings show that FGCI works well for small sample case because the FGPQ of ${\sigma }_{i}^{2}$ might contain some weak points that affect the FGPQ of *μ*_*i*_ as the sample case increases. For large sample sizes, MOVER was the best method for small *σ*^2^, which is possibly caused by the CI for ${\mu }_{i}+{\sigma }_{i}^{2}$. Meanwhile, the next best one was PB, which has the strong point of using a resampling technique to collect information about several populations even when the variance *σ*^2^ is large.

**Table 5:** Daily rainfall data in five Thailand’s regions on August 5, 2019.

Northern		Northeastern							Central		Eastern	Southern			
3	0	3	0	0	49.5	0	0	0	2.9	3.2	0	4.1	0	0	2.7
2.6	5	0	40	1.5	10.5	0	0	0	0.2	0	3.2	0	0	0	0
1	23.8	0	3.5	18.5	60.4	4	0	11	0.3	0	10.4	11.5	3.5	0	0
3.6	16	0	0	42	12.7	0	0	0	2.5	4.7	1.1	2.5	13.6	0	0
0	11.5	0	12	9.1	6.8	0	20.3	0	0.4	19.3	0.2	9.7	0	0.2	0
13.2	1.2	0	15	6	69.3	0	0	0	0.4	3.1	4.3	10.4	0	0	0
22.4	10.3	0	0	7.5	36.5	0	2.4	0.3	1.1	2.9	0	9.6	0	0	0
1.4	1.7		0	1.5	0	8.6	0	0	1	0	5.7	0 19	0	0	0
18.3	5.5	0	0.7	6.3	0	0	0	0	1.3	0.9	0	8.3	0	0	0
0	7.3	0	0	0	0	0	0	0	0.1	0	0	0	4.8	0	6.2
15.5	24.3	1.7	3	0.4	0	0	0	0	2.9	0	0.2	0	0	0	0
0	27.2	2.3	0	0	3.8	0	0	0	0	2.6	0.1	0	0	0	0
0	12.6	0.5	0	0	0	0	3.2	0	1	17	62.8	0	0	0	6.1
0	22.7	3.9	0	0	0	0	0	0	4.7	0	36.7	17.8	0	0	0
9.8	0	6.9	29.4	1.8	0	0	0	0	0.5	3.5	15.6	12.3	0	0	0
24.3	2.6	2.2	48	0	0	0	0	0	5	0	50	2.5	0	0	0
24.6	0	3.2	0	0	0	6	0	0	2.5	0	35.5	0	0	0	0.3
8.8	3.2	5.3	70.8	14.3	0	0	0	0	0	0	35	0.9	0	0	0
0	2.6	11	3.5	0	0	0	0	0	0	5.1	5.9	0	0	0	0
19.8	2	0.6	14.2	0	0	0	4.8	0	0	60.4	0	2.6	0	0	0
5	8	0	7	0	0	2.3	0	0	0	6.9	0	0	0	0	0
12.3	1.9	1	0	0	21.5	0	0	0	6.6	3	3	0	0	0	0
8.1	0.8	2.4	0	0	2.5	1	0	0	0	15.1	60.4	2	0	0	0
4.8	2.2	13.2	0	0	0	0	0	0	9.5	6	60	0	0	0	0
5.8	6.5	0.4	0	0	13	0	0		5.1	13.4	76	0	0	0	0
17	0	0	10.8	0	26.2	0	0		12.5	6.2	79.7	0	0	0	0
25.1	2.2	1.3	0	10.1	2.2	4.6	5.4		0		65.7	3.5	0	0	
8.3	0	10	6.3	0	3	0	0		0		108	0	0	36.1	
22.9	4.3	2.5	0	4.8	10.5	10			0	3.2	10.5	0	0	41.8	
26.9	0.2	4.6	4	0	0	0	12		0			0	0	30	
0	0	0	19.3	0	0	9.5	0				2.2	0	0	0	

**Notes.**

Source: Thai Meteorological Department: https://www.tmd.go.th/services/weekly report.php.

**Table 6:** Daily rainfall data in five Thailand’s regions on August 9, 2019.

Northern		Northeastern							Central		Eastern	Southern			
9.5	0	25.3	20	6.6	8.4	0	67	0	39.6	0	0	27.9	4.1	0.4	114.6
4.9	10	25.5	14.5	16.9	0.8	2.9	65.4	0	25	0	0	0	9	3.8	0
0	21.6	24	3	10	20.2	0	21	0	0	0	26.5	3.4	27.3	0.6	0
4.7	15	8	28	48.2	0	14.3	6.4	7.2	0	0	36.4	0	6.5	0	0
0	15.5	0	27	6.5	0.5	0	0	3.5	29.7	0.1	0	0.8	3.5	10.8	0
63.2	14	20	50	4.8	5.3	6	52	0	0	0.3	4.5	37.9	0	5	18.2
9.6	8.5	0	24	25	16.7	0	45	40.5	3.1	0.5	0	32.4	0	12.2	40.4
10.7	11.5	0	30	0	45.2	28	41.4	25.8	8.2	31.5	0.5	33.8	0	3.6	0
13	17.4	0	22	0	0	0	14.3	30.4	3.2	8.2	0.7	15.8	0	0	0
0	15.6	0	16	3.2	0.6	0	45	0	7.1	0	12.3	0	3.6	8.8	10.8
0	31.6	33.8	0	44	0	0	27	0	0	0	0.5	0	3	0	0
0	20.6	33.7	0	0	3.1	27.6	0.2	0	3.2	0	1.9	0	1	0	0
0	31.1	15.1	0	9.3	33.3	33	30	0	4.2	0	66.4	0	3.7	6.2	35
0	16.3	18.5	0	0	6	0	0	0	5.7	0	93.6	11.5	15.6	0	0
2.8	0	44.8	39.7	20	0	0	0	8.3	30	0	68.7	1.7	11.2	3.8	33.5
11.3	33.1	37.5	9.3	0	13.2	0	0	0	4	0	40	1.2	24	0	57
0.6	29.2	0	0	4.8	0	0	0	0	0	0	65	21.2	0	0	10.5
36.1	11.2	47	2.1	0	21	0	0	0	0	0	63.7	0	0	0	0
0	14.4	20	0	0	0	0	1	36.1	0	0	9.2	30	10.2	0.2	0
2.6	60	30.8	46.7	0	8.4	15	0	0	0	1.2	0	5.1	0	0	0
5	42.3	30	10.5	0	0	0	0	12.5	0	0	0	2.5	0	0	30.8
13.4	9.5	1	0	56.5	0	0	2.5	0	14.7	0.1	11	2.4	0	0.4	10.7
12.3	34.5	1.2	41	39.2	0.5	0	0	0	0	1	69.6	5	0	0	0
25.8	36.5	56.3	10.3	0	4.5	25.7	9.5	0	0	3	89.6	1.7	0	0	15.9
30.2	9.7	0	1.2	6.4	16.2	41.4	0	0	0.5	160		0	0	2.2	0
16.4	0	6	23.9	5.3	0	41.6	0		1.6	34.3	0	0	0
6	0	0	22.2	0	3.5	53.8	0		0		0	2.1	0	0.6	
33.1	7.6	5.3	24.1	9.8	20	48.5	0		0		25	0	0	76.6	
16.4	9.6	7.2	38	0	0	78.5	2.1		0		19.5	10.5	7	121.6	
19.8	9.3	24.6	9	9.7	0	12.7		0	0			15.3	10.6	60	
0	0	30	9.2	4.5	1.2	80.9	0		0			0	3.6	0	

**Notes.**

Source: Thai Meteorological Department: https://www.tmd.go.th/services/weekly_report.php.

**Figure 4:**
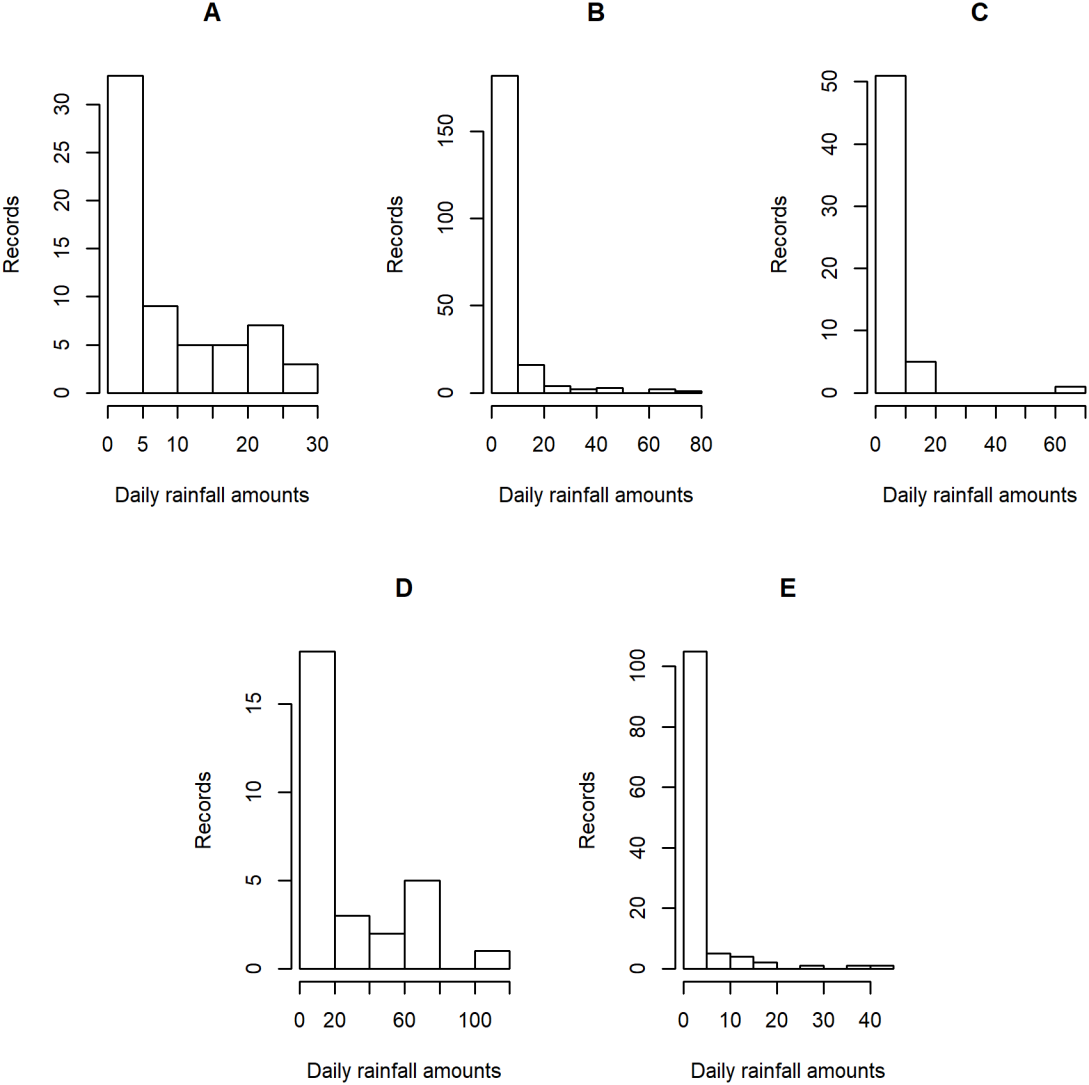
Histogram plots of daily rainfall data in five Thailand’s regions on August 5, 2019: (A) Northern (B) Northeastern (C) Central (D) Eastern (E) Southern.

**Figure 5:**
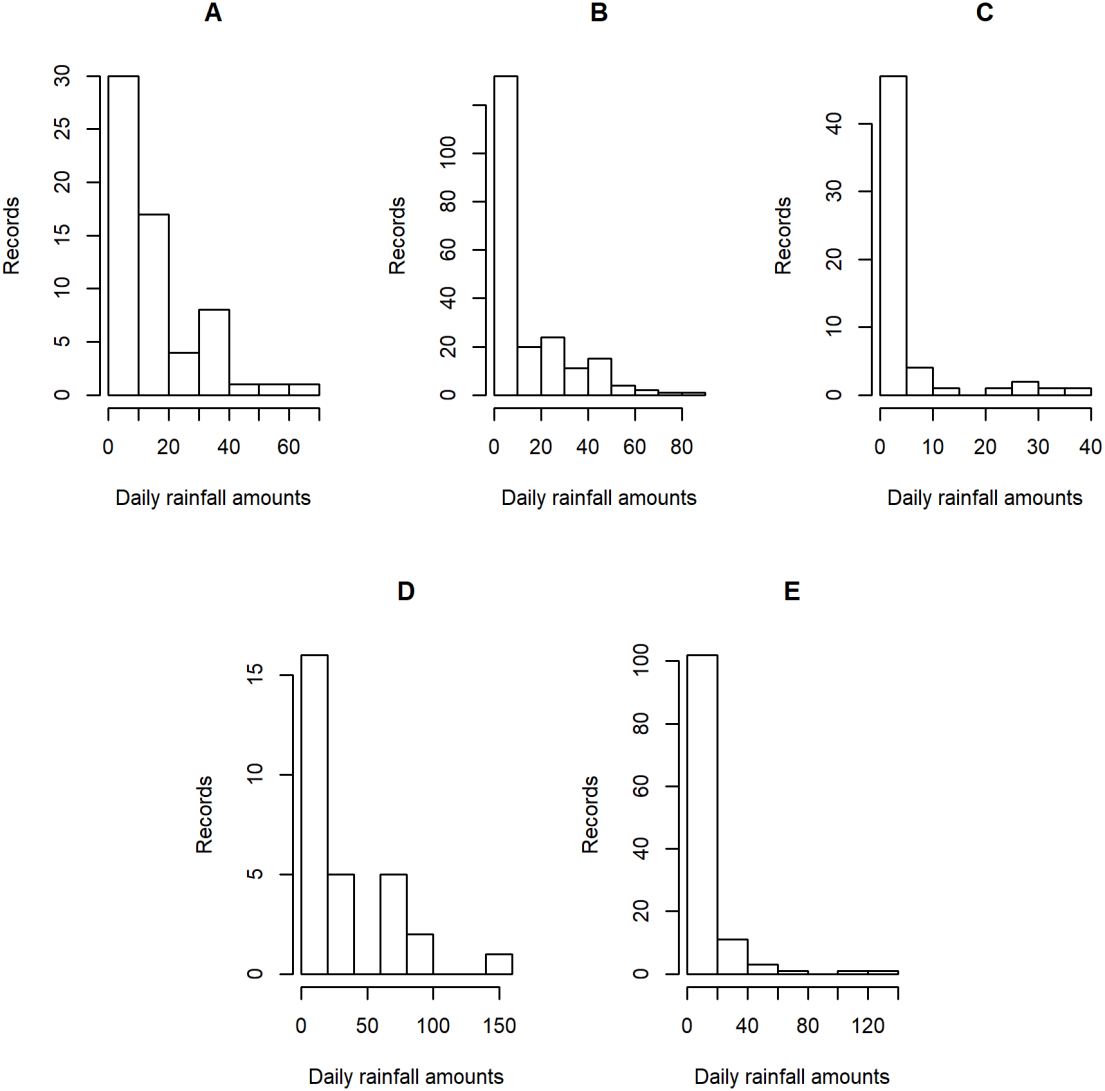
Histogram plots of daily rainfall data in five Thailand’s regions on August 9, 2019: (A) Northern (B) Northeastern (C) Central (D) Eastern (E) Southern.

**Figure 6:**
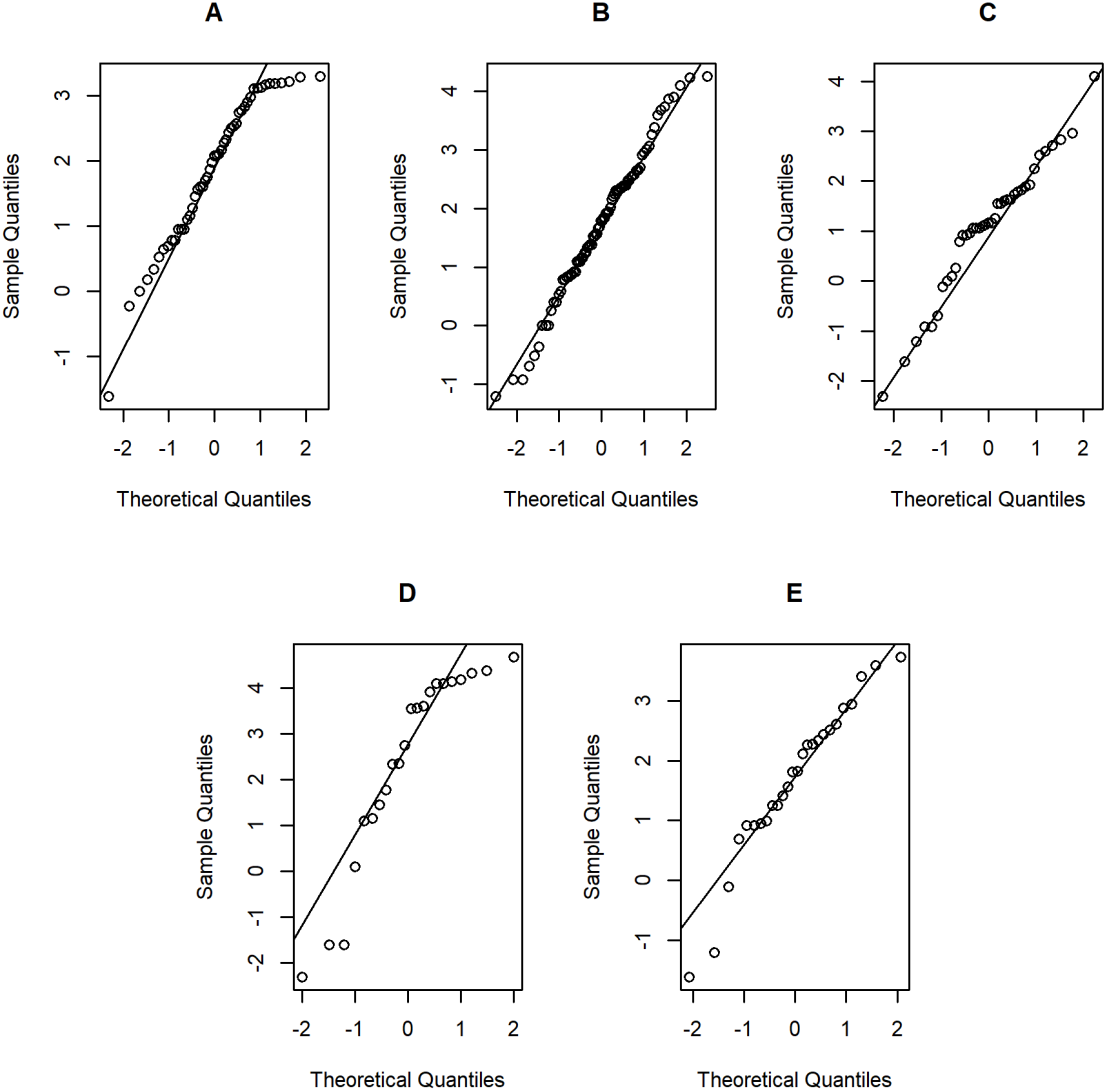
Normal Q-Q plots of log-positive daily rainfall data in five Thailand’s regions on August 5, 2019: (A) Northern (B) Northeastern (C) Central (D) Eastern (E) Southern.

## An empirical application

Daily rainfall data obtained from the Thai Meteorological Department (TMD) were divided into the northern, northeastern, central, and eastern regions, while the southern region was a combination of the data from the southeastern and southwestern shores. Due to the differences in the climate patterns and meteorological conditions in the five regions, we focused was on estimating the daily rainfall data in these regions by treating them as separate sets of observations rather than using the average rainfall for the whole of Thailand by pooling them and treating them as a single population. The daily rainfall amounts were recorded on August 5 and 9, 2019, which is in the middle of the rainy season (mid-May to mid-October) when rice farming is conducted in Thailand. Entries with rainfall of less than 0.1 mm were considered as zero records.

**Figure 7:**
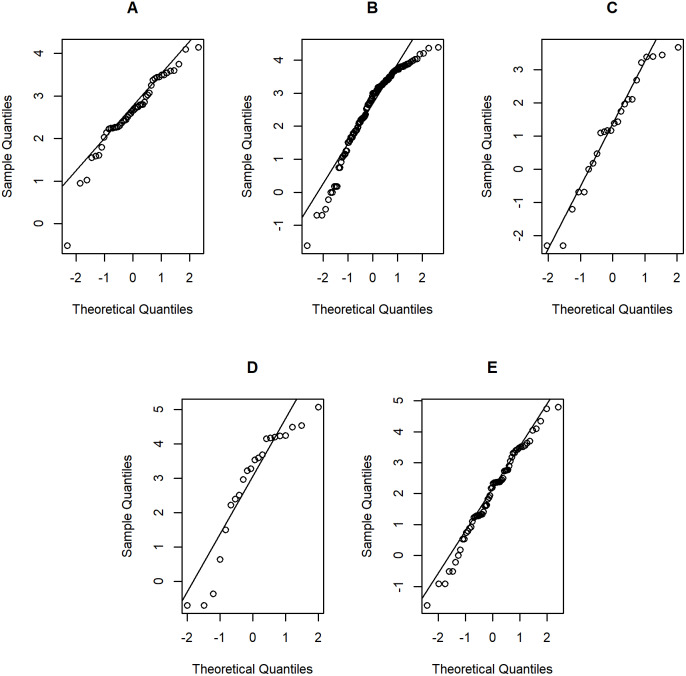
Normal Q-Q plots of log-positive daily rainfall data in five Thailand’s regions on August 9, 2019: (A) Northern (B) Northeastern (C) Central (D) Eastern (E) Southern.

Tables 5–6 contain the daily rainfall records for the five regions, while Figs. 4–5 show histogram plots of rainfall observations, and Figs. 6–7 exhibit normal Q-Q plots of the log-positive rainfall data on August 5 and 9, 2019, respectively. It can be seen that the data for all of the regions contained zero observations. After that, the fitted distribution of the positive observations was checked using the Akaike information criterion (AIC), as reported in Table 7. It can be concluded that the rainfall data in all of the regions on August 5 and 9, 2019 follow a delta-lognormal distribution. All data sets and R code are available in the Supplemental Information. The summary statistics are reported in Table 8. In the approximation of the daily rainfall amounts in the five regions, the estimated common means were 4.4506 and 13.2621 mm/day on August 5 and 9, 2019, respectively. The computed 95% CIs of the common rainfall mean are reported in Table 9. Under the rain criteria issued by the TMD (Department, 2018), it can be interpreted that the daily rainfall in Thailand on August 5, 2019, was light (0.1–10.0 mm), while it was moderate (10.1–35.0 mm) on August 9, 2019. These results confirm the simulation results for *k* = 5 in the previous section.

**Table 7:** AIC results of daily rainfall records in five Thailand’s regions.

Regions	AIC				
	Cauchy	Logistic	Lognormal	Normal	T-distribution
On August 5, 2019					
Northern	373.1958	357.3122	**336.8724**	353.7757	354.3055
Northeastern	600.9473	642.1779	**543.9619**	667.2334	664.6152
Central	240.0227	266.4162	**220.8503**	293.9151	283.2302
Eastern	229.8995	220.2523	**202.8394**	218.7240	219.1471
Southern	194.9368	197.5586	**178.5587**	201.1654	200.1388
On August 9, 2019					
Northern	389.6257	387.3072	**375.7994**	391.1802	390.2479
Northeastern	1123.7491	1080.8694	**1052.8953**	1080.1467	1079.9365
Central	178.8516	189.5353	**155.0261**	190.6855	190.5103
Eastern	233.5236	227.1725	**215.9306**	228.0501	227.4559
Southern	541.0477	569.2615	**487.4667**	592.2242	588.2377

**Table 8:** The summary statistics.

Regions	Estimated parameters				
	*n*_*i*_	${\hat {\mu }}_{i}$	${\hat {\sigma }}_{i}^{2}$	${\hat {\delta }}_{i}$	${\hat {\vartheta }}_{i}$
August 5, 2020					
Northern	62	1.866	1.277	0.210	9.472
Northeastern	210	1.734	1.578	0.619	4.668
Central	57	1.085	1.784	0.316	4.741
Eastern	29	2.366	4.545	0.241	59.391
Southern	119	1.684	1.730	0.782	2.639
August 9, 2020					
Northern	62	2.621	0.732	0.226	15.187
Northeastern	210	2.577	1.502	0.405	16.429
Central	57	1.190	3.054	0.579	5.542
Eastern	29	2.860	3.070	0.241	52.813
Southern	119	2.007	2.051	0.462	10.811

**Table 9:** 95%CIs of common rainfall mean in five Thailand’s regions.

Methods	95%CIs for *ϑ*		Lengths
	Lower	Upper	
On August 5, 2020			
FGCI	2.5545	6.3342	3.7798
LS	3.2166	5.6846	2.4681
MOVER	2.7216	9.0296	6.3080
PB	5.8876	11.4965	5.6089
HPD-JR	3.5216	7.8533	4.3317
HPD-NGB	2.4969	6.0904	3.5935
On August 9, 2020			
FGCI	7.1127	16.8809	9.7682
LS	10.4880	16.0363	5.5483
MOVER	7.5814	23.3171	15.7357
PB	14.5229	23.5821	9.0591
HPD-JR	12.8404	20.4349	7.5945
HPD-NGB	7.2928	17.1265	9.8337

## Discussion

It can be seen that for MOVER and PB developed from the studies of Krishnamoorthy & Oral (2015) and Malekzadeh & Kharrati-Kopaei (2019), respectively, the simulation results are similar to both of these studies provided that the zero observations are omitted. CIs for the common mean have been investigated in both normal and lognormal distributions (Fairweather, 1972; Jordan & Krishnamoorthy, 1996; Krishnamoorthy & Mathew, 2003; Lin & Lee, 2005; Tian & Wu, 2007; Krishnamoorthy & Oral, 2015). However, the common mean of delta-lognormal populations is especially of interest because it can be used to fit the data from real-world situations such as investigating medical costs (Zou, Taleban & Huo, 2009; Tierney et al., 2003; Tian, 2005), analyzing airborne contaminants (Owen & DeRouen, 1980; Tian, 2005) and measuring fish abundance (Fletcher, 2008; Wu & Hsieh, 2014). Furthermore, it is possible that some extreme rainfall data also fulfill the assumptions of a delta-lognormal distribution. Note that such natural disasters as floods and landslides have been caused by the extreme rainfall events, as evidenced in many country around the world: Europe (e.g., Northern England, Southern Scotland and Ireland Otto & Oldenborgh, 2017), Asia (e.g., Japan Oldenborgh, 2018) and North America (e.g., Southeast Texas Oldenborgh et al., 2019). Our findings show that some of the methods studied had CPs that were too low or too high for large sample cases, a shortcoming that should be addressed in future work.

## Conclusions

The objective of this study was to propose CIs for the common mean of several delta-lognormal distributions using FGCI, LS, MOVER, PB, HPD-JR, and HPD-NGB. The CP and AL as performance measures of the methods were assessed via Monte Carlo simulation. The findings confirm that for small sample case ()k=2 (), FGCI and HPD-NGB are the recommended methods in different situations: FGCI (a small-to-moderate sample size and a large ${\sigma }_{i}^{2}$ with a moderate-to-large sample size) and HPD-NGB (small ${\sigma }_{i}^{2}$ with a moderate-to-large sample size). For large sample cases (*k* = 5, 10), MOVER small *δ*_*i*_ and ${\sigma }_{i}^{2}$) and PB (large *δ*_*i*_ and ${\sigma }_{i}^{2}$) performed the best.

##  Supplemental Information

10.7717/peerj.10758/supp-1 Supplemental Information 1 Daily rainfall data in five Thailand’s regions on August 5, 2019

10.7717/peerj.10758/supp-2 Supplemental Information 2 Daily rainfall data in five Thailand’s regions on August 9, 2019

10.7717/peerj.10758/supp-3 Supplemental Information 3 R code for the program for running all outputs.

## Additional Information and Declarations

### Data Availability

The following information was supplied regarding data availability: All data sets and R code are available in the Supplementary Files.
