# Supplementary material for: Estimating the average daily rainfall in Thailand using confidence intervals for the common mean of several delta-lognormal distributions
Source: PeerJ. 2021 Jan 22;9:e10758. doi: 10.7717/peerj.10758 (PMC7831370; doi:10.7717/peerj.10758)
Supplement: Supplemental Information 1 [file peerj-09-10758-s001.docx]

**Dataset S1**. Daily rainfall data in five Thailand's regions on August 5, 2019

| **Northern** | |  | **Northeastern** | | | | | | |  | **Central** | |  | **Eastern** |  | **Southern** | | | |
| --- | --- | --- | --- | --- | --- | --- | --- | --- | --- | --- | --- | --- | --- | --- | --- | --- | --- | --- | --- |
| 3 | 0 |  | 3 | 0 | 0 | 49.5 | 0 | 0 | 0 |  | 2.9 | 3.2 |  | 0 |  | 4.1 | 0 | 0 | 2.7 |
| 2.6 | 5 |  | 0 | 40 | 1.5 | 10.5 | 0 | 0 | 0 |  | 0.2 | 0 |  | 3.2 |  | 0 | 0 | 0 | 0 |
| 1 | 23.8 |  | 0 | 3.5 | 18.5 | 60.4 | 4 | 0 | 11 |  | 0.3 | 0 |  | 10.4 |  | 11.5 | 3.5 | 0 | 0 |
| 3.6 | 16 |  | 0 | 0 | 42 | 12.7 | 0 | 0 | 0 |  | 2.5 | 4.7 |  | 1.1 |  | 2.5 | 13.6 | 0 | 0 |
| 0 | 11.5 |  | 0 | 12 | 9.1 | 6.8 | 0 | 20.3 | 0 |  | 0.4 | 19.3 |  | 0.2 |  | 9.7 | 0 | 0.2 | 0 |
| 13.2 | 1.2 |  | 0 | 15 | 6 | 69.3 | 0 | 0 | 0 |  | 0.4 | 3.1 |  | 4.3 |  | 10.4 | 0 | 0 | 0 |
| 22.4 | 10.3 |  | 0 | 0 | 7.5 | 36.5 | 0 | 2.4 | 0.3 |  | 1.1 | 2.9 |  | 0 |  | 9.6 | 0 | 0 | 0 |
| 1.4 | 1.7 |  | 0 | 1.5 | 0 | 8.6 | 0 | 0 | 1 |  | 0 | 5.7 |  | 0 |  | 19 | 0 | 0 | 0 |
| 18.3 | 5.5 |  | 0 | 0.7 | 6.3 | 0 | 0 | 0 | 0 |  | 1.3 | 0.9 |  | 0 |  | 8.3 | 0 | 0 | 0 |
| 0 | 7.3 |  | 0 | 0 | 0 | 0 | 0 | 0 | 0 |  | 0.1 | 0 |  | 0 |  | 0 | 4.8 | 0 | 6.2 |
| 15.5 | 24.3 |  | 1.7 | 3 | 0.4 | 0 | 0 | 0 | 0 |  | 2.9 | 0 |  | 0.2 |  | 0 | 0 | 0 | 0 |
| 0 | 27.2 |  | 2.3 | 0 | 0 | 3.8 | 0 | 0 | 0 |  | 0 | 2.6 |  | 0.1 |  | 0 | 0 | 0 | 0 |
| 0 | 12.6 |  | 0.5 | 0 | 0 | 0 | 0 | 3.2 | 0 |  | 1 | 17 |  | 62.8 |  | 0 | 0 | 0 | 6.1 |
| 0 | 22.7 |  | 3.9 | 0 | 0 | 0 | 0 | 0 | 0 |  | 4.7 | 0 |  | 36.7 |  | 17.8 | 0 | 0 | 0 |
| 9.8 | 0 |  | 6.9 | 29.4 | 1.8 | 0 | 0 | 0 | 0 |  | 0.5 | 3.5 |  | 15.6 |  | 12.3 | 0 | 0 | 0 |
| 24.3 | 2.6 |  | 2.2 | 48 | 0 | 0 | 0 | 0 | 0 |  | 5 | 0 |  | 50 |  | 2.5 | 0 | 0 | 0 |
| 24.6 | 0 |  | 3.2 | 0 | 0 | 0 | 6 | 0 | 0 |  | 2.5 | 0 |  | 35.5 |  | 0 | 0 | 0 | 0.3 |
| 8.8 | 3.2 |  | 5.3 | 70.8 | 14.3 | 0 | 0 | 0 | 0 |  | 0 | 0 |  | 35 |  | 0.9 | 0 | 0 | 0 |
| 0 | 2.6 |  | 11 | 3.5 | 0 | 0 | 0 | 0 | 0 |  | 0 | 5.1 |  | 5.9 |  | 0 | 0 | 0 | 0 |
| 19.8 | 2 |  | 0.6 | 14.2 | 0 | 0 | 0 | 4.8 | 0 |  | 0 | 60.4 |  | 0 |  | 2.6 | 0 | 0 | 0 |
| 5 | 8 |  | 0 | 7 | 0 | 0 | 2.3 | 0 | 0 |  | 0 | 6.9 |  | 0 |  | 0 | 0 | 0 | 0 |
| 12.3 | 1.9 |  | 1 | 0 | 0 | 21.5 | 0 | 0 | 0 |  | 6.6 | 3 |  | 3 |  | 0 | 0 | 0 | 0 |
| 8.1 | 0.8 |  | 2.4 | 0 | 0 | 2.5 | 1 | 0 | 0 |  | 0 | 15.1 |  | 60.4 |  | 2 | 0 | 0 | 0 |
| 4.8 | 2.2 |  | 13.2 | 0 | 0 | 0 | 0 | 0 | 0 |  | 9.5 | 6 |  | 60 |  | 0 | 0 | 0 | 0 |
| 5.8 | 6.5 |  | 0.4 | 0 | 0 | 13 | 0 | 0 |  |  | 5.1 | 13.4 |  | 76 |  | 0 | 0 | 0 | 0 |
| 17 | 0 |  | 0 | 10.8 | 0 | 26.2 | 0 | 0 |  |  | 12.5 | 6.2 |  | 79.7 |  | 0 | 0 | 0 | 0 |
| 25.1 | 2.2 |  | 1.3 | 0 | 10.1 | 2.2 | 4.6 | 5.4 |  |  | 0 |  |  | 65.7 |  | 3.5 | 0 | 0 |  |
| 8.3 | 0 |  | 10 | 6.3 | 0 | 3 | 0 | 0 |  |  | 0 |  |  | 108 |  | 0 | 0 | 36.1 |  |
| 22.9 | 4.3 |  | 2.5 | 0 | 4.8 | 10.5 | 10 | 0 |  |  | 3.2 |  |  | 10.5 |  | 0 | 0 | 41.8 |  |
| 26.9 | 0.2 |  | 4.6 | 4 | 0 | 0 | 0 | 12 |  |  | 0 |  |  |  |  | 0 | 0 | 30 |  |
| 0 | 0 |  | 0 | 19.3 | 0 | 0 | 9.5 | 0 |  |  | 2.2 |  |  |  |  | 0 | 0 | 0 |  |
| Source: Thai Meteorological Department | | | | | | | | | | | | | | | | | | | |
| <https://www.tmd.go.th/services/weekly_report.php> | | | | | | | | | | | | | | | | | | | |
