# Supplementary material for: Estimating the average daily rainfall in Thailand using confidence intervals for the common mean of several delta-lognormal distributions
Source: PeerJ. 2021 Jan 22;9:e10758. doi: 10.7717/peerj.10758 (PMC7831370; doi:10.7717/peerj.10758)
Supplement: Supplemental Information 2 [file peerj-09-10758-s002.docx]

**Dataset S2**. Daily rainfall data in five Thailand's regions on August 9, 2019

| **Northern** | |  | **Northeastern** | | | | | | |  | **Central** | |  | **Eastern** |  | **Southern** | | | |
| --- | --- | --- | --- | --- | --- | --- | --- | --- | --- | --- | --- | --- | --- | --- | --- | --- | --- | --- | --- |
| 9.5 | 0 |  | 25.3 | 20 | 6.6 | 8.4 | 0 | 67 | 0 |  | 39.6 | 0 |  | 0 |  | 27.9 | 4.1 | 0.4 | 114.6 |
| 4.9 | 10 |  | 25.5 | 14.5 | 16.9 | 0.8 | 2.9 | 65.4 | 0 |  | 25 | 0 |  | 0 |  | 0 | 9 | 3.8 | 0 |
| 0 | 21.6 |  | 24 | 3 | 10 | 20.2 | 0 | 21 | 0 |  | 0 | 0 |  | 26.5 |  | 3.4 | 27.3 | 0.6 | 0 |
| 4.7 | 15 |  | 8 | 28 | 48.2 | 0 | 14.3 | 6.4 | 7.2 |  | 0 | 0 |  | 36.4 |  | 0 | 6.5 | 0 | 0 |
| 0 | 15.5 |  | 0 | 27 | 6.5 | 0.5 | 0 | 0 | 3.5 |  | 29.7 | 0.1 |  | 0 |  | 0.8 | 3.5 | 10.8 | 0 |
| 63.2 | 14 |  | 20 | 50 | 4.8 | 5.3 | 6 | 52 | 0 |  | 0 | 0.3 |  | 4.5 |  | 37.9 | 0 | 5 | 18.2 |
| 9.6 | 8.5 |  | 0 | 24 | 25 | 16.7 | 0 | 45 | 40.5 |  | 3.1 | 0.5 |  | 0 |  | 32.4 | 0 | 12.2 | 40.4 |
| 10.7 | 11.5 |  | 0 | 30 | 0 | 45.2 | 28 | 41.4 | 25.8 |  | 8.2 | 31.5 |  | 0.5 |  | 33.8 | 0 | 3.6 | 0 |
| 13 | 17.4 |  | 0 | 22 | 0 | 0 | 0 | 14.3 | 30.4 |  | 3.2 | 8.2 |  | 0.7 |  | 15.8 | 0 | 0 | 0 |
| 0 | 15.6 |  | 0 | 16 | 3.2 | 0.6 | 0 | 45 | 0 |  | 7.1 | 0 |  | 12.3 |  | 0 | 3.6 | 8.8 | 10.8 |
| 0 | 31.6 |  | 33.8 | 0 | 44 | 0 | 0 | 27 | 0 |  | 0 | 0 |  | 0.5 |  | 0 | 3 | 0 | 0 |
| 0 | 20.6 |  | 33.7 | 0 | 0 | 3.1 | 27.6 | 0.2 | 0 |  | 3.2 | 0 |  | 1.9 |  | 0 | 1 | 0 | 0 |
| 0 | 31.1 |  | 15.1 | 0 | 9.3 | 33.3 | 33 | 30 | 0 |  | 4.2 | 0 |  | 66.4 |  | 0 | 3.7 | 6.2 | 35 |
| 0 | 16.3 |  | 18.5 | 0 | 0 | 6 | 0 | 0 | 0 |  | 5.7 | 0 |  | 93.6 |  | 11.5 | 15.6 | 0 | 0 |
| 2.8 | 0 |  | 44.8 | 39.7 | 20 | 0 | 0 | 0 | 8.3 |  | 30 | 0 |  | 68.7 |  | 1.7 | 11.2 | 3.8 | 33.5 |
| 11.3 | 33.1 |  | 37.5 | 9.3 | 0 | 13.2 | 0 | 0 | 0 |  | 4 | 0 |  | 40 |  | 1.2 | 24 | 0 | 57 |
| 0.6 | 29.2 |  | 0 | 0 | 4.8 | 0 | 0 | 0 | 0 |  | 0 | 0 |  | 65 |  | 21.2 | 0 | 0 | 10.5 |
| 36.1 | 11.2 |  | 47 | 2.1 | 0 | 21 | 0 | 0 | 0 |  | 0 | 0 |  | 63.7 |  | 0 | 0 | 0 | 0 |
| 0 | 14.4 |  | 20 | 0 | 0 | 0 | 0 | 1 | 36.1 |  | 0 | 0 |  | 9.2 |  | 30 | 10.2 | 0.2 | 0 |
| 2.6 | 60 |  | 30.8 | 46.7 | 0 | 8.4 | 15 | 0 | 0 |  | 0 | 1.2 |  | 0 |  | 5.1 | 0 | 0 | 0 |
| 5 | 42.3 |  | 30 | 10.5 | 0 | 0 | 0 | 0 | 12.5 |  | 0 | 0 |  | 0 |  | 2.5 | 0 | 0 | 30.8 |
| 13.4 | 9.5 |  | 1 | 0 | 56.5 | 0 | 0 | 2.5 | 0 |  | 14.7 | 0.1 |  | 11 |  | 2.4 | 0 | 0.4 | 10.7 |
| 12.3 | 34.5 |  | 1.2 | 41 | 39.2 | 0.5 | 0 | 0 | 0 |  | 0 | 1 |  | 69.6 |  | 5 | 0 | 0 | 0 |
| 25.8 | 36.5 |  | 56.3 | 10.3 | 0 | 4.5 | 25.7 | 9.5 | 0 |  | 0 | 3 |  | 89.6 |  | 1.7 | 0 | 0 | 15.9 |
| 30.2 | 9.7 |  | 0 | 1.2 | 6.4 | 16.2 | 41.4 | 0 |  |  | 0 | 0.5 |  | 160 |  | 0 | 0 | 2.2 | 0 |
| 16.4 | 0 |  | 6 | 23.9 | 5.3 | 0 | 41.6 | 0 |  |  | 0 | 1.6 |  | 34.3 |  | 0 | 0 | 0 | 0 |
| 6 | 0 |  | 0 | 22.2 | 0 | 3.5 | 53.8 | 0 |  |  | 0 |  |  | 0 |  | 2.1 | 0 | 0.6 |  |
| 33.1 | 7.6 |  | 5.3 | 24.1 | 9.8 | 20 | 48.5 | 0 |  |  | 0 |  |  | 25 |  | 0 | 0 | 76.6 |  |
| 16.4 | 9.6 |  | 7.2 | 38 | 0 | 0 | 78.5 | 2.1 |  |  | 0 |  |  | 19.5 |  | 10.5 | 7 | 121.6 |  |
| 19.8 | 9.3 |  | 24.6 | 9 | 9.7 | 0 | 12.7 | 0 |  |  | 0 |  |  |  |  | 15.3 | 10.6 | 60 |  |
| 0 | 0 |  | 30 | 9.2 | 4.5 | 1.2 | 80.9 | 0 |  |  | 0 |  |  |  |  | 0 | 3.6 | 0 |  |
| Source: Thai Meteorological Department | | | | | | | | | | | | | | | | | | | |
| <https://www.tmd.go.th/services/weekly_report.php> | | | | | | | | | | | | | | | | | | | |
